# The *C. elegans* gene *gvd-1* promotes late larval development and germ cell proliferation

**DOI:** 10.1242/bio.059978

**Published:** 2023-06-30

**Authors:** Anbalagan Pon Ezhil Buvani, Kuppuswamy Subramaniam

**Affiliations:** ^1^ Department of Biotechnology, Indian Institute of Technology–Madras, Chennai 600036, India; ^2^ Department of Biological Sciences & Bioengineering, Indian Institute of Technology, Kanpur 208016, India

**Keywords:** *puf-8*, Postembryonic development, Vulva, Seam cells, Sheath cells

## Abstract

Limiting maternal resources necessitates deferring the development of adult-specific structures, notably the reproductive structures, to the postembryonic phase. These structures form postembryonically from blast cells generated during embryogenesis. A close coordination of developmental timing and pattern among the various postembryonic cell lineages is essential to form a functional adult. Here, we show that the *C. elegans* gene *gvd-1* is essential for the development of several structures that form during the late larval stages. In *gvd-1* mutant animals, blast cells that normally divide during the late larval stages (L3 and L4) fail to divide. In addition, germ cell proliferation is also severely reduced in these animals. Expression patterns of relevant reporter transgenes revealed a delay in G1/S transition in the vulval precursor cell P6.p and cytokinesis failure in seam cells in *gvd-1* larvae. Our analyses of GVD-1::GFP transgenes indicate that GVD-1 is expressed in both soma and germ line, and functions in both. Sequence comparisons revealed that the sequence of *gvd-1* is conserved only among nematodes, which does not support a broadly conserved housekeeping function for *gvd-1*. Instead, our results indicate a crucial role for *gvd-1* that is specific to the larval development of nematodes.

## Teaser

**Summary:** We show that *gvd-1*, a nematode-specific *C. elegans* gene is essential for cell division in diverse postembryonic cell lineages, including the germ line, during late larval stages.

## INTRODUCTION

In many animal species, not all organs are generated during embryogenesis. Some adult structures, which are not essential for the juvenile to feed and grow, develop postembryonically from blast cells formed during embryogenesis. These blast cells enter temporary cell-cycle quiescence as soon as they are formed and reenter the cell cycle in response to environmental cues such as the availability of food, and systemic cues such as the growth and development of other structures. In the nematode *Caenorhabditis elegans*, during postembryonic development, blast cells in multiple lineages resume the cell cycle at different larval stages and form new structures such as the vulva. These features make this organism a great model to investigate the pathways that transduce and integrate the various cues that control postembryonic development.

In *C. elegans*, embryogenesis generates two primordial germ cells (PGCs) and 53 somatic blast cells (SBCs), which resume the cell cycle during larval stages (Sulston and Horvitz, 1977). At the time of hatching, the mitotic cell cycle is paused in PGCs at the G2 phase and in SBCs at the G1 phase (Park and Krause, 1999; Boxem and Van Den Heuvel, 2001; Fukuyama et al., 2003, 2006). Feeding stimulates growth and cell division in the newly hatched L1 larva. In the absence of food, neither PGCs nor SBCs resume the cell cycle, and L1 larvae can remain in a developmentally arrested state, known as L1 diapause, for several weeks (Castro et al., 2012). Activation of the insulin/insulin-like growth factor (IGF) signaling pathway is essential for larval development induced by the nutritional cue (Baugh and Sternberg, 2006; Baugh, 2013). Mutations in the sole IGF receptor, DAF-2, lead to L1 arrest even in the presence of food, and PGCs fail to undergo starvation-induced cell-cycle arrest when the IGF pathway antagonist DAF-18/PTEN is mutated (Gems et al., 1998; Fukuyama et al., 2006). At least one mechanism that links the IGF signaling with the cell-cycle reentry has been established in some SBCs that resume the cell cycle at the L1 stage. The cell cycle is paused in these SBCs by expression of the cyclin-dependent kinase inhibitor CKI-1. Genetic studies indicate that the IGF pathway, when activated, suppresses CKI-1 expression by inactivating the DAF-16/FOXO transcription factor, which otherwise promotes CKI-1 expression (Baugh and Sternberg, 2006).

Feeding alone is not sufficient to activate all the postembryonic cell divisions. For example, while the PGCs begin to divide almost immediately after the newly hatched L1 larva starts to feed, SBCs such as the P lineage cells P1-P12 divide only at mid-L1 and the G2 cell divides only at mid-L2 (Sulston and Horvitz, 1977). Furthermore, in some lineages, whereas one of the two daughter cells produced by the blast cell generates more descendants without further temporary cell-cycle arrest, its sibling may remain quiescent for extended periods of time. This is well exemplified in the P lineage: whereas the anterior descendants of P3-P8 cells continue to divide during the L1 stage and generate neurons and glial cells, the posterior descendants do not divide until the mid-L3 stage (Sulston and Horvitz, 1977). Clearly, cues other than the initial availability of food, probably of systemic nature arising from multiple lineages, are likely crucial for coordinating the cell cycle and development during late larval stages. These cues, and how the development is coordinated among the different lineages have not been well understood.

Here, we describe the phenotypic defects caused by a potential null allele of a nematode-specific gene, named *gvd-1* (*germ line and vulva defective*). Cell divisions that occur during L2/L3 transition and during L3 stage are defective *gvd-1* in animals. In *gvd-1* hermaphrodites, these defects affect the development of the vulva, spermatheca, uterus, gonadal sheath cells, seam, and the germ line. In males, formation of the tail rays, which arise from the seam cell lineage is defective. Expression and rescue analyses using transgene reporters indicate that GVD-1 is expressed in the above lineages, with predominant nuclear localization, and functions both in the germ line and soma. Thus, our results uncover an important role for *gvd-1* in the developmental events that occur in multiple lineages during late larval stages.

## RESULTS

### *kp20*, a mutant allele of *gvd-1*, causes synthetic-sterile phenotype with mutation in *puf-8*

**Fig. 1.**
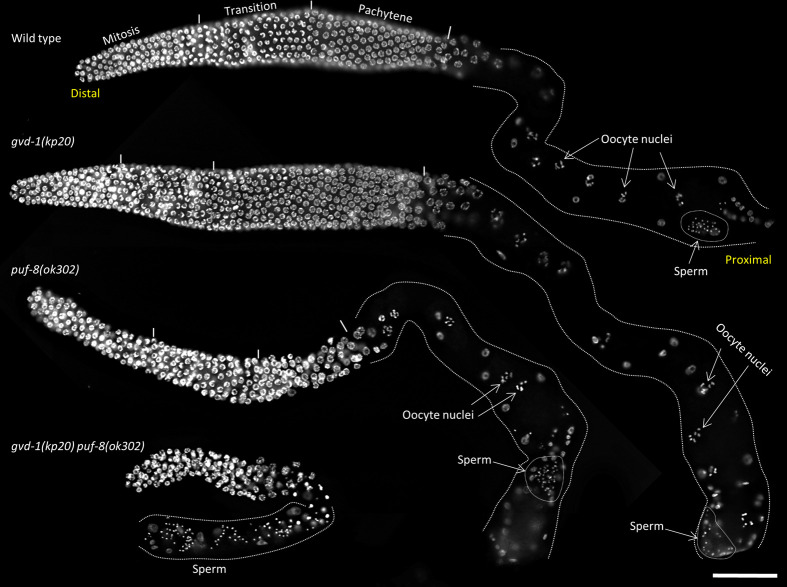
**Reduction of *gvd-1* function displays synthetic germline phenotypes with *puf-8* mutation.** Extruded germ lines of the indicated genotypes stained with DAPI are shown. The different developmental stages are marked with short vertical lines and labelled on the top of the wild-type germ line. The proximal region has been outlined in white dotted lines. Note: oocyte nuclei are seen in the wild type, and *gvd-1* and *puf-8* single mutants, but not in *gvd-1 puf-8* double mutant. Scale bar: 50 μm.

Mutant *C. elegans* strains lacking the conserved RNA-binding protein PUF-8 are fertile at 20°C, but sterile at 25°C (Subramaniam and Seydoux, 2003). To identify genes that potentially compensated PUF-8's absence at 20°C, a genetic screen had been carried out earlier in our laboratory. Several new alleles in other genes that cause synthetic-sterile phenotype with *puf-8(-)* were isolated in this screen. These new mutant alleles do not cause any obvious phenotype on their own but lead to sterility even at 20°C when in double mutant combination with *puf-8(-)* (Vaid et al., 2013). Here, we present our results on one such mutant allele named *kp20*. The germ lines of *kp20/kp20* hermaphrodites resemble the wild type, produce both sperm and oocytes, and yield viable progeny. By contrast, the germ lines of animals homozygous for *ok302*, a null allele of *puf-8*, and *kp20* are drastically smaller than both the single mutants and produce no oocytes (Fig. 1). Through two-factor genetic crosses, SNP-mapping, genome-sequencing, and RNA-mediated interference (RNAi), we have identified *kp20* as an allele of a novel gene with the sequence name *W10D9.6* (www.wormbase.org) (see Materials and Methods for details). Based on the phenotype of a potential null allele (see below), we have named the new gene as *gvd-1* (*germ line and vulva defective-1*).

*gvd-1* locus has been predicted to encode two protein isoforms. Isoform-a consists of 178 amino acids (aa), and isoform-b consists of only the last 97 aa of isoform-a (www.wormbase.org), hereafter referred to as *gvd-1A* and *gvd-1B*, respectively. The *kp20* allele is a G-to-A substitution that replaces an aspartic acid (position 124 in GVD-1A and 43 in GVD-1B) with asparagine; the aspartic acid at this position is conserved in several nematode genera (Figs S4, S5). Sequence comparisons identified potential orthologs of *gvd-1* only in the nematode phyla (Fig. S5). However, domain search analysis at InterPro (http://www.ebi.ac.uk/interpro/) using InterProScan revealed that GVD-1 belongs to a family of proteins that contain a domain of unknown function called DUF4796 typified by a human protein called MKNR2 opposite strand protein (MKRN2OS; accession H3BPM6), whose function is unknown. This domain encompasses the entire 178 aa of GVD-1A. A potential paralog of *gvd-1*, called *W10D9.3*, shares high sequence similarity with the potential orthologs present in other organisms including MKRN2OS (Figs S6, S7 and S8). Although the amino acid sequences of GVD-1 and W10D9.3 are not similar, the exon-intron organization, the proximity between the two loci – about 900 bp of intervening sequence – and the remarkable conservation of the nucleotide sequences of intron-2 (Fig. S9) suggest that *gvd-1* and *W0D9.3* might have arisen through gene duplication, and the coding sequence of *gvd-1* might have diverged substantially from the conserved *W10D9.3*. Consistent with the sequence divergence, we did not see any functional redundancy between *gvd-1* and *W10D9.3* (see below).

### *kp20* is a reduction-of-function allele of *gvd-1*

To test whether *kp20* is a null allele, we depleted GVD-1 in *gvd-1(kp20)* animals using RNAi. The *kp20* allele delays larval development: while wild-type animals reached adulthood in about 72 h post-egg laying at 20°C, the *gvd-1(kp20)* animals required 96 h to reach adulthood (Fig. S10). By contrast, 70% of the RNAi-treated *gvd-1(kp20)* animals reached adulthood only at about 120 h and 30% of them remained arrested at the L4 stage (Fig. S10). The dsRNA chosen here targets both the GVD-1 isoforms, but not *W10D9.3*. Additionally, *kp91*, a potential null allele of *W10D9.3* (Fig. S11) did not cause any observable phenotypic defects. Furthermore, the RNAi-treatment caused only a mild effect on the wild-type animals. Therefore, it is unlikely that the more severe phenotype observed in the RNAi-treated *gvd-1(kp20)* animals resulted from an off-target effect of RNAi. Instead, the observed phenotype most likely resulted from the combined effect of the *kp20* mutation on GVD-1 function and the RNAi-mediated depletion of GVD-1. It is possible that the *kp20* mutation partially compromises GVD-1 activity, and the RNAi reduces the levels of, but does not eliminate, GVD-1. Thus, *kp20* is potentially a reduction-of-function allele of *gvd-1*.

### Animals homozygous for *kp86*, a putative null allele of *gvd-1A*, grow more slowly than the wild type and are sterile

**Fig. 2.**
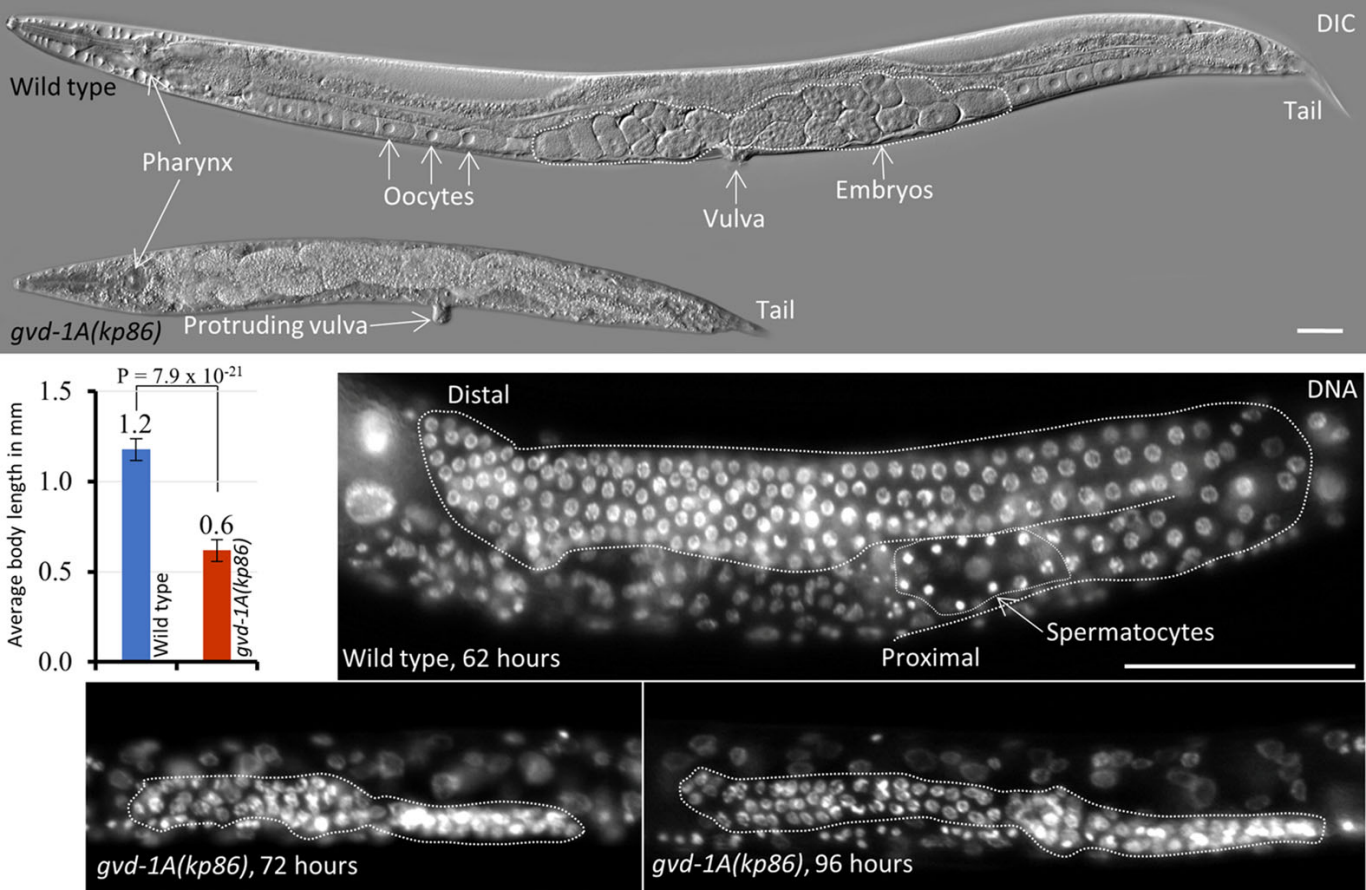
**Potential null allele of *gvd-1A*, *kp86*, causes growth, vulval and germline defects.** (Top) wild-type and *gvd-1A(kp86)* adult animals visualized using differential interference contrast (DIC) microscopy are shown. (Middle left) quantification of the body lengths of wild-type and *gvd-1A(kp86)* adults. (Middle right, bottom) sections of wild-type and *gvd-1A(86)* larvae stained with DAPI at the indicated time points are shown. The germ line is outlined with white dotted lines in all three images. *gvd-1A(kp86)* animals form protruding vulva and have substantially smaller body (top) and germ line than the wild-type (compare middle and bottom images). Scale bar: 50 μm.

To determine the phenotypic consequences of complete loss of *gvd-1A* function, we generated *kp86*, a potential null allele, by engineering a frameshift mutation that shifts and truncates the reading frame after the 12th aa of GVD-1A using the CRISPR/Cas9 method (Fig. S12). The *kp86* allele does not affect GVD-1B. Thus, the phenotypic defects described here result from the loss of isoform-a alone. *gvd-1* is located at map position −15.61 on chromosome II, and this map position is not covered by any of the available genetic balancers that suppress recombination. Due to this, we maintained *kp86*-carrying strains as *gvd-1A(kp86)* / *cdc-37(tm2403)* trans heterozygotes. *cdc-37(tm2403)* is located at −15.59 and causes larval-arrest phenotype in homozygous condition. In contrast to *gvd-1(kp20)* animals, which were all fertile, the *gvd-1A(kp86)* animals were all sterile (*n*=500) and formed protruding vulva (Fig. 2). In addition, the growth rate of *gvd-1A(kp86)* larvae was drastically reduced: at 72 h post-egg laying, when all wild-type animals had become adults, none of the *gvd-1A(kp86)* animals reached adulthood (Fig. S10). Even at the adult stage, the mutant animals were about half the size of the wild type (Fig. 2).

### *gvd-1A* promotes germ cell proliferation

*gvd-1A(kp86)* germ lines contained far fewer germ cells than the wild-type and did not produce sperm or oocytes. In wild-type animals, the number of germ cells rapidly increases during the L3 and L4 stages and reaches to about 1000 germ cells per gonadal arm in the adult (Kimble and White, 1981). By contrast, in *gvd-1A(kp86)* animals, there were only about 30 germ cells per gonadal arm (Fig. 2; Fig. S13). Furthermore, *gvd-1A(kp86)* suppressed the over proliferation of germ cells observed in *glp-1(ar202)* germ lines in which germ cells fail to initiate meiosis, and in GLD-1-depleted germ lines in which the female germ cells prematurely exit meiosis and return to mitotic cycling (Fig. S14) (Francis et al., 1995; Pepper et al., 2003). These observations indicate that *gvd-1A* is generally required for the mitotic division of germ cells. Additionally, the few germ cells present in *gvd-1A(kp86)* animals did not become sperm (Fig. 2), which indicates that the proliferation defect observed in these animals is not due to premature differentiation.

**Fig. 3.**
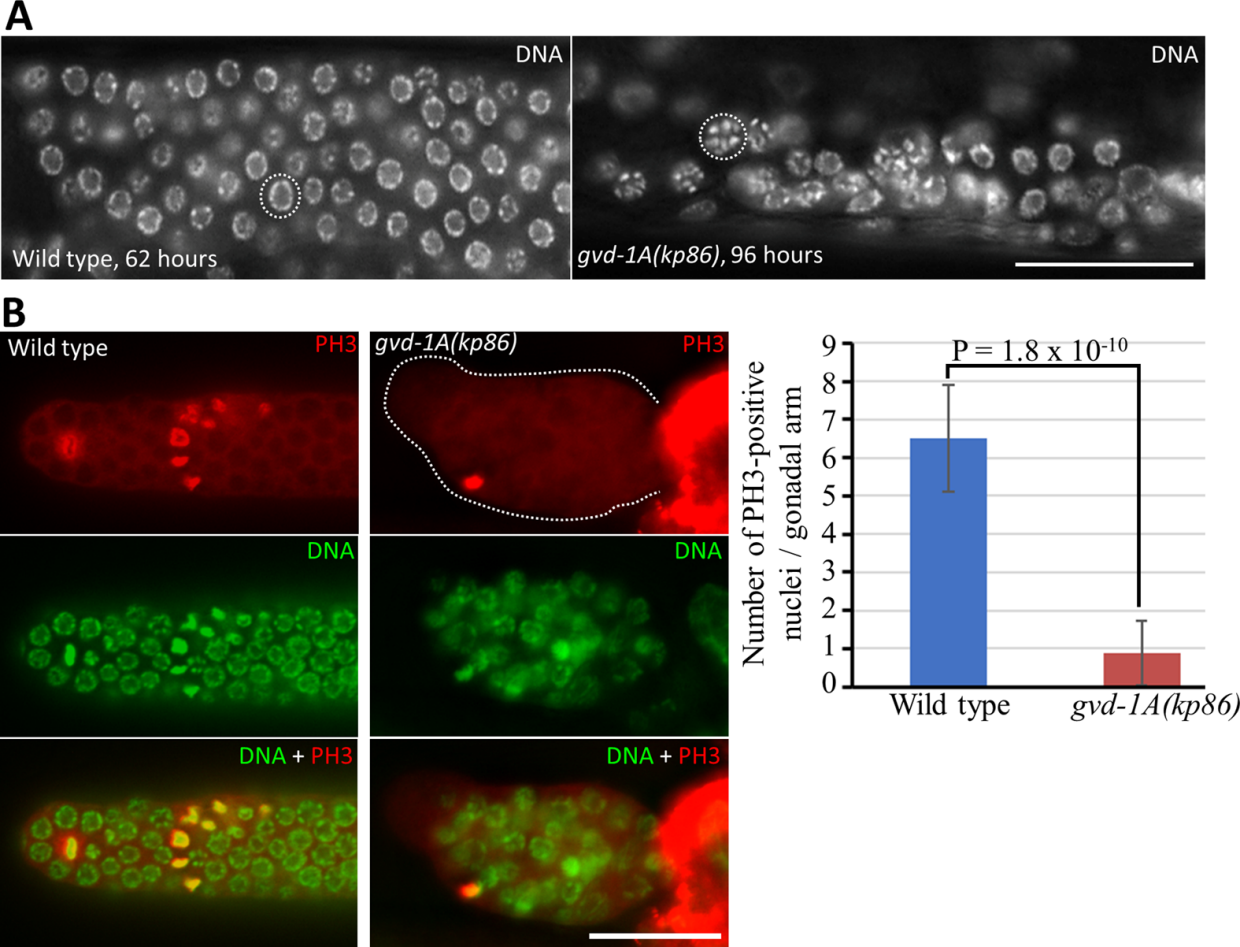
**Mutations in *gvd-1* affect germ cell chromatin morphology.** (A) Distal part of the germ line in whole animals stained with DAPI are shown. Chromatin in *gvd-1A(kp86)* germ line appear condensed (compare the nuclei outlined in dotted circles between the two genotypes). (B) Dissected germ lines stained with both anti-PH3 antibody and DAPI. The bar graph shows the average number of PH3-positive nuclei / gonadal arm. Error bars represent standard deviations, and the *P* values were calculated by two-tailed Student's *t*-test. Scale bar: 50 μm.

The chromatin of *gvd-1A(kp86)* germ cells, as revealed by DAPI-staining, appeared condensed and resembled the chromatin at prometaphase (Fig. 3A). To test if these cells were indeed arrested at prometaphase, we stained the wild-type and *gvd-1A(kp86)* germ lines with the G2/M marker phospho-histone H3 (PH3). Contrary to our expectation, we could detect only an occasional PH3-positive nucleus in a few *gvd-1A(kp86)* germ lines, which indicates that the *gvd-1A(kp86)* germ nuclei are not arrested at G2/M (Fig. 3B).

### Cell divisions in the somatic gonadal SS lineage during late larval stages require *gvd-1*

**Fig. 4.**
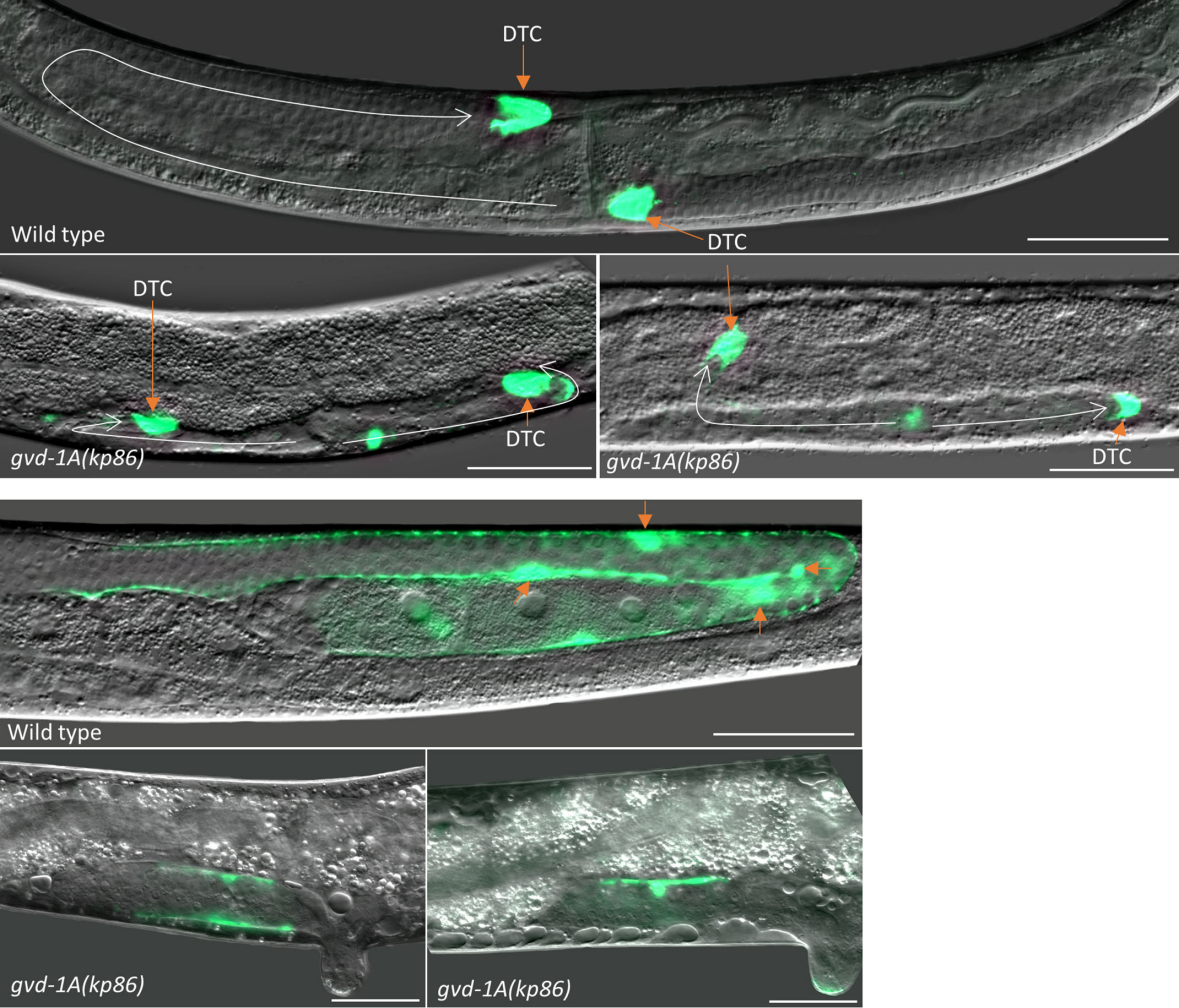
**Migration of the distal tip cells and divisions of the sheath cells are defective in *gvd-1A(kp86)* animals.** Merged DIC and GFP fluorescence images of parts of young adults of the indicated genotypes are shown. DTCs (A) and sheath cells (B) have been visualized using *lag-2p*:GFP and *lim-7p*:GFP transgenes, respectively. (A) In the wild type, both DTCs have migrated to their normal position, approximately at the center of the body. By contrast, in the two images of *gvd-1A(kp86)* animals shown, both DTCs have migrated only a short distance. Approximate routes of migration are indicated by arrows. (B) The cell bodies of one each of the four pairs (Sh1-Sh4) of sheath cells in the wild type are indicated by arrows. Long, mesh-like cytoplasmic extension of Sh1 is clearly visible in the wild-type image. By contrast, only one or two Sh cells with very little cytoplasmic extension are seen in the two *gvd-1A(kp86)* larvae shown here. Scale bar: 50 μm.

Next, we focused on the somatic gonad as germ cell and vulval defects often accompany defective development of the somatic gonad (Seydoux et al., 1993; Killian and Hubbard, 2005). For this, we examined the morphology of different somatic gonadal structures in *gvd-1A(kp86)* hermaphrodites using cell-specific markers. The entire somatic gonad is derived from two founder cells called Z1 and Z4. These two cells produce 12 descendants that form the somatic gonadal primordium (SGP) by the time of second larval molt (L2/L3 molt). During L3, germ cells and somatic gonadal cells rearrange such that two somatic cells (Z1/Z4 granddaughters), called the distal tip cells (DTCs), are located one each at the anterior and posterior ends of the gonad. The remaining 10 Z1/Z4 descendants form the central part with germ cells located between the central part and the DTCs. The DTCs migrate away from the center and lead gonadal elongation, and eventually form the germline stem cell (GSC) niche. The 10 centrally located somatic cells divide further and produce the gonadal sheath, spermatheca and uterus (Kimble and Hirsh, 1979). First, we examined the formation and migration of DTCs with the help of *lag-2* promoter-driven GFP expression (*lag-2p*:GFP), which marks the DTCs (Blelloch et al., 1999). Both DTCs were formed in *gvd-1A(kp86)* animals, but they failed to complete the migration. In some animals, DTCs turn back abruptly without migrating to the dorsal side, and in a few others, they managed to migrate dorsally and form the characteristic loop but failed to migrate further. In a few other animals, the DTCs remained on the ventral side forming short gonadal arms (Fig. 4). While these results show that *gvd-1A* is not required for DTC specification, we are not certain that the observed migration defect was directly caused by the loss of *gvd-1A* function, as the migration defect could have been caused by the lack of germ cell proliferation.

**Fig. 5.**
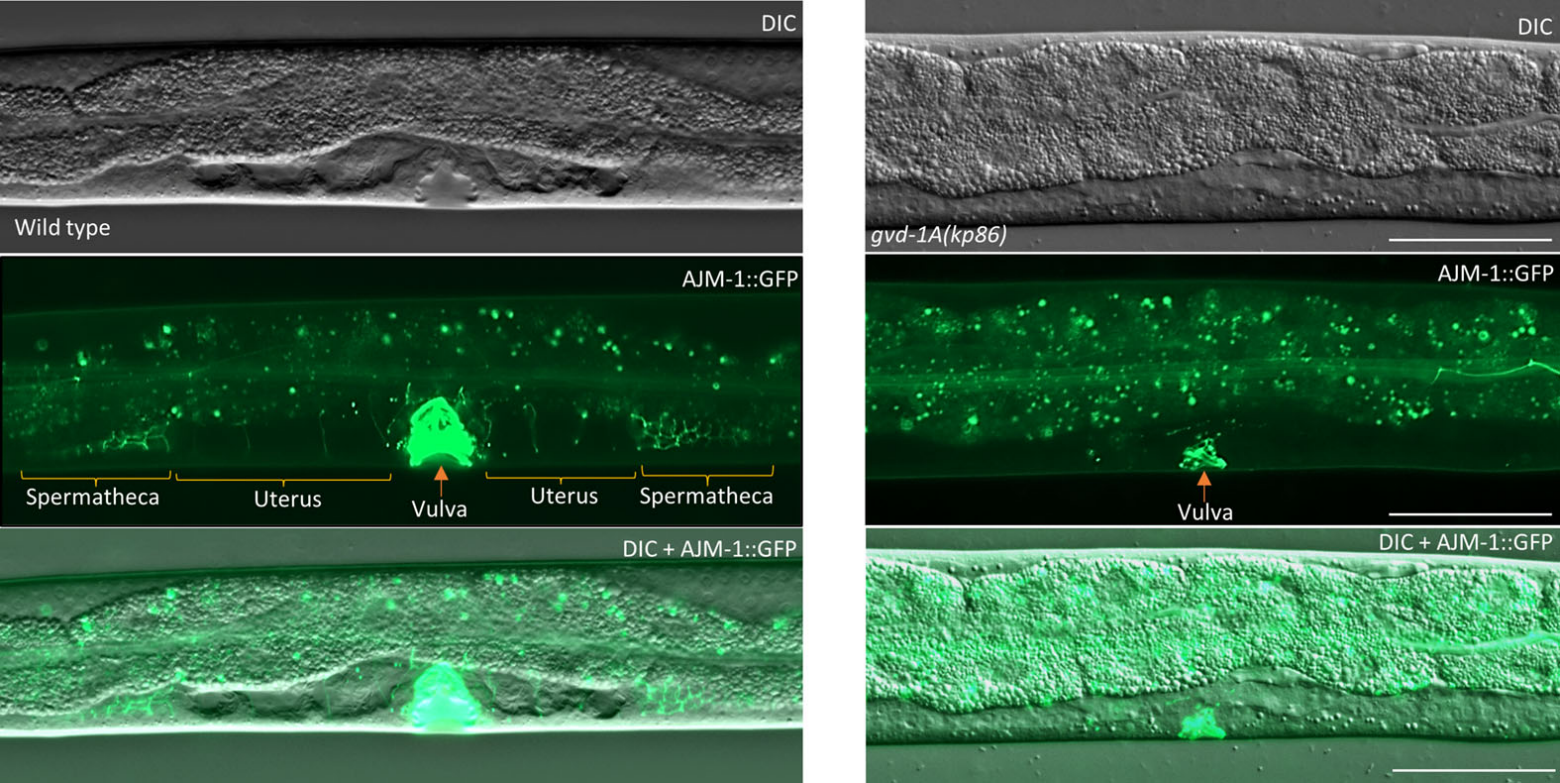
**The spermatheca and uterus are defective in *gvd-1A(kp86)* animals.** The central parts of wild-type and *gvd-1A(kp86)* L4 larvae are shown. In the wild type, the adherens junction marker AJM-1::GFP expression is seen on the cell membranes of spermatheca and uterus (apart from strong expression in vulva) (Koppen et al., 2001). By contrast, in *gvd-1A(kp86)* larvae, AJM-1::GFP expression is absent on either side of the vulva where spermatheca and uterus are normally present. Scale bar: 50 μm.

The gonadal sheath, which envelops germ cells in most part of the germ line, consists of five pairs of sheath cells (Sh1-Sh5 pairs) per gonadal arm. All five Sh pairs arise from four blast cells called SS cells (spermatheca/sheath) after the L2/L3 molt (Kimble and Hirsh, 1979; Mccarter et al., 1997). We tracked the formation and development of Sh cells using *lim-7p*:GFP which marks the Sh1-Sh4 pairs (Hall et al., 1999). In wild type, Sh1 cells expand their cytoplasm distally and form a mesh-like structure that envelopes the germ cells. By contrast, we did not see any such cytoplasmic extension or meshwork in *gvd-1A(kp86)* germ lines. In addition, these germ lines had fewer Sh cells: while we could detect eight Sh cells per arm in wild-type larvae, there were only two Sh cells in *gvd-1A(kp86)* larvae (Fig. 4; Fig. S15). These results indicate that *gvd-1A* is essential for cell divisions in the SS lineage. Presumably, the absence of cytoplasmic extensions resulted from the failure to produce Sh1 cells. Consistently, the spermatheca, a major part of which also consists of SS descendants, was severely defective in *gvd-1A(kp86)* animals, although we did not determine if there were any reduction in the number of spermatheca cells (Fig. 5). The first two rounds of cell divisions in the Z1/Z4 lineages appeared to be unaffected in *gvd-1A(kp86)* animals as we could detect the DTCs and at least two Sh cells, which are normally produced at the second round of Z1/Z4 divisions, in these animals. In addition, we found that the formation of anchor cell (AC), which is produced at the third round, was also unaffected in *gvd-1A(kp86)* animals (Fig. S16). The third round of Z1/Z4 divisions completes the formation of the 12-cell SGP. Thus, these results show that *gvd-1A* is not essential for cell fate specification and for cell divisions in the SS lineage until the formation of SGP but is required for the cell divisions after that.

### *gvd-1A* promotes the division of the vulval precursor cell P6.p and its descendants

**Fig. 6.**
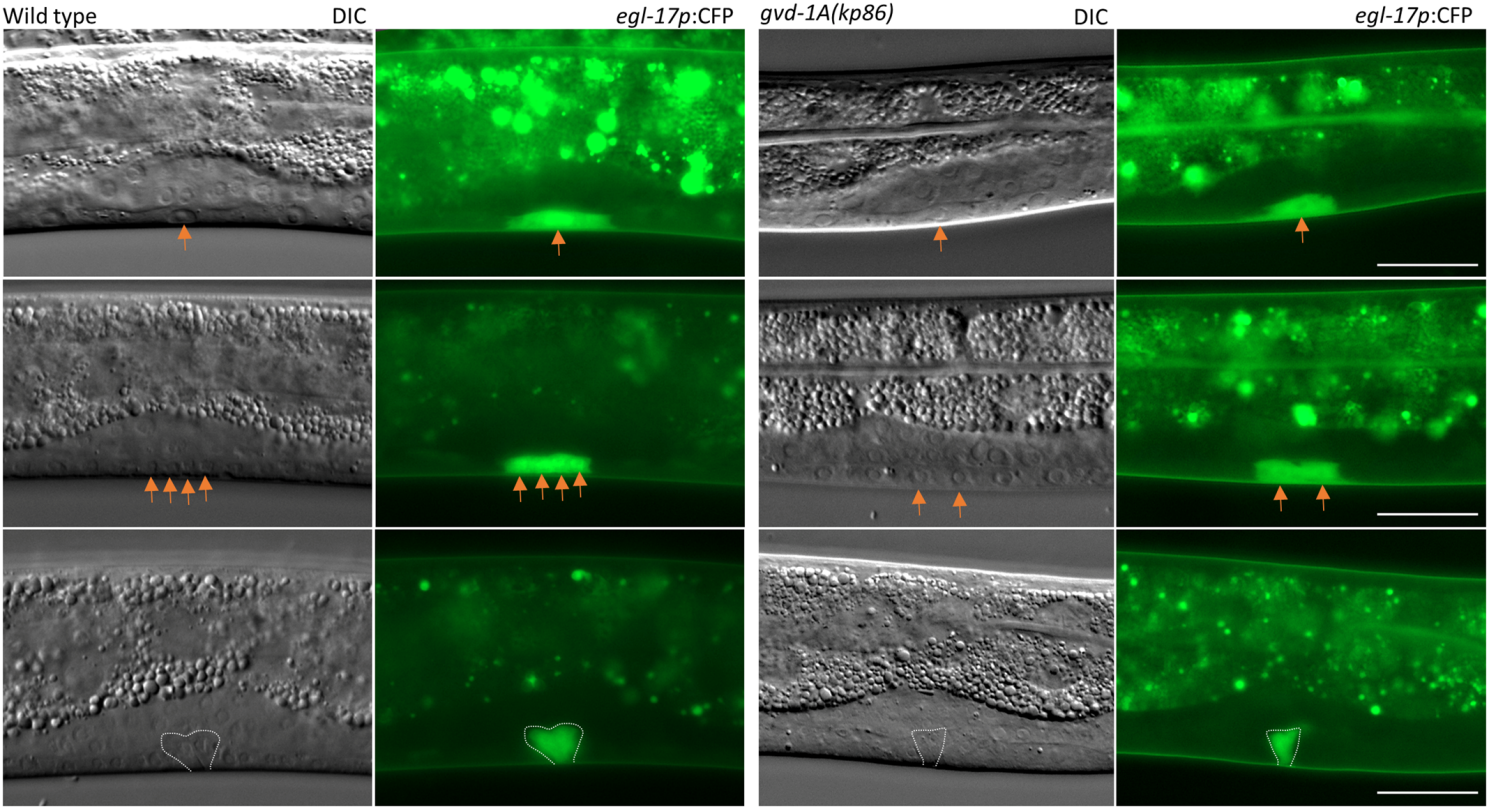
**Descendants of the vulval precursor cell P6.p fail to divide in *gvd-1A(kp86)* larvae.** The central parts of larvae, in which P6.p and its descendants have been visualized using *egl-17p*:GFP (arrows) are shown. The wild-type larvae shown on the top, middle and bottom were imaged at 40-, 50- and 60-h post-egg laying, respectively. The *gvd-1A(kp86)* larvae were imaged at 68-, 84- and 96-h post-egg laying. The wild-type P6.p divides twice and generates four cells by 50 h post-egg laying. By contrast, the first division of *gvd-1A(kp86)* P6.p is considerably delayed, and the second division does not occur. However, the P6.p descendants in these larvae undergo shape changes characteristic of invasion (Bottom images). Scale bar: 20 μm. For quantitation, see Fig. S17.

In *gvd-1A(kp86)* adult hermaphrodites, a protruding structure was prominent where the vulva is normally located. In wild-type hermaphrodites, vulva is formed by the progeny of three ventral hypodermal cells named P5.p, P6.p and P7.p. Between the L2/L3 and L3/L4 molts, P6.p divides three times and generates eight cells (Sulston and Horvitz, 1977). We tracked the first two of these divisions using *egl-17p*:CFP, which marks the P6.p and its descendants (Inoue et al., 2002). As expected, a single cell expressing *egl-17p*:CFP – P6.p – could be observed at about 40 h post-egg laying in the wild type, which divided twice generating two cells and then four cells that were arranged in a row at the ventral side. These divisions were completed by about 50 h. By contrast, even at 70 h, about 58% of *gvd-1A(kp86)* larvae had only one *egl-17p*:CFP-positive cell. Two *egl-17p*:CFP-positive cells could be observed in the remaining 42%, but none had four *egl-17p:GFP*-positive cells (Fig. 6; Fig. S17). These results reveal a requirement of *gvd-1* for the division of P6.p. Since *gvd-1A(kp86)* larvae were indistinguishable from the wild type at earlier time points, we could not determine if P6.p was specified at the normal time in these larvae. Despite the cell division defects described above, the two P6.p descendants present in some *gvd-1A(kp86)* larvae began differentiation, marked by abnormal invagination (Fig. 6; Fig. S18), which is consistent with the earlier observation that the cell division and differentiation are separable events during vulva formation (Fay and Han, 2000).

### *gvd-1* may be dispensable for embryonic and early larval development

Somatic gonadal structures and the vulva are generated from blast cells during postembryonic development; the PGCs as well begin to proliferate only during this phase. This prompted us to explore if *gvd-1A* was involved in the formation of other structures that develop postembryonically. First, we wished to determine whether the postembryonic cell divisions that occur during the L1 stage were affected by the loss of *gvd-1A* function. For this, we examined the cell divisions in mesodermal and germ lineages in which the precursors begin to divide during the L1 stage. The mesodermal precursor M cell undergoes four rounds of divisions during L1 stage (Sulston and Horvitz, 1977). Using a *hlh-8* promoter-driven GFP reporter, which expresses in M and its descendants (Harfe et al., 1998), we examined these divisions and found them to be unaffected in *gvd-1A(kp86)* larvae (Fig. S19). Similarly, the early divisions of primordial germ cells (PGCs) Z2 and Z3 were also unaffected in *gvd-1A(kp86)* larvae (Fig. S19). These are consistent with what we observed in the SS lineage (see above). Furthermore, *gvd-1A(kp86)* animals did not exhibit uncoordinated locomotion, which is characteristic of mutations that affect the divisions of ventral nerve cord precursors at the L1 stage (O'Connell et al., 1998). Since the *gvd-1A(kp86)* larvae examined here were the progenies of *gvd-1A(kp86/+)* heterozygotes, it is possible that the maternal GVD-1 perdured until the late L1 stage. To test if the maternally provided GVD-1 functioned in embryos and early-stage larvae, we resorted to RNAi by dsRNA injection into the germ lines of young adults – a strategy that has been shown to deplete both maternal and zygotic mRNA (Fire et al., 1998). Injection of *gvd-1*-specific dsRNA into wild-type or *gvd-1A(kp86/+)* heterozygous animals did not cause embryonic lethality. Importantly, it did not phenocopy the larval defects caused by the *kp86* mutation, indicating that the RNAi was ineffective in these animals. As an alternative, we injected *gvd-1A(kp86/kp86)* animals that were partially rescued by the presence of both *kpIs7* and *kpIs106* transgenes (see below) with the dsRNA. Embryos produced by these animals were viable and hatched into larvae that did not display any locomotion defects but, like the *gvd-1A(kp86)* larvae, formed protruding vulva and developed into sterile adults (Fig. S20). These observations suggest that GVD-1 is not crucial for embryonic and early larval development. Nevertheless, we cannot rule out the possibility that low levels of maternal GVD-1 that might have remained even after RNAi was sufficient for the early development.

### *gvd-1* is essential for seam cell development in both hermaphrodites and males

**Fig. 7.**
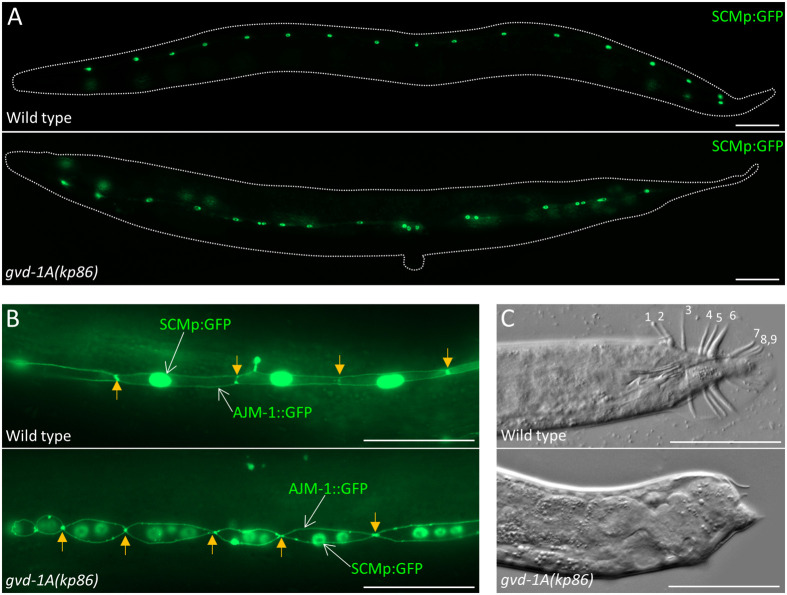
**Aberrant seam cell cytokinesis and absence of male-tail rays in *gvd-1A(kp86)* animals.** (A) Seam cell nuclei have been visualized using SCMp:GFP transgene in adult hermaphrodites. In the wild type, seam cell nuclei are evenly positioned along the length of the body, whereas they are unevenly positioned in the mutant. (B) Sections of L4 larvae in which the seam cell nuclei and cell membranes are visualized using SCMp:GFP and AJM-1::GFP are shown. Arrows point to junctions between adjacent seam cells. In contrast to the wild type, in which only one nucleus is seen in each cell, up to three nuclei per cell are visible in the mutant. (C) DIC images of wild-type and *gvd-1A(kp86)* male tails are shown. The rays, numbered on the wild-type image, are absent in the mutant. Scale bar: 50 μm.

Next, we turned our attention to the seam cell lineage in which cells continue to divide at all larval stages (Sulston and Horvitz, 1977; Altun and Hall, 2009). The seam cell nuclei can be readily recognized using the SCMp:GFP marker (Terns et al., 1997). A newly hatched larva contains 10 pairs of seam cells. After stem-like divisions and proliferation by some of them during L1-L4, these cells ultimately form 16 cells on either side at the late L4 stage, which then fuse into a syncytium with 16 nuclei at the adult stage. As expected, we were able to observe evenly spaced 16 nuclei on the lateral sides of wild-type adults. By contrast, in *gvd-1A(kp86)* adults, seam cell nuclei were randomly positioned. In some animals, they were far apart, and in the others, multiple nuclei were clustered together (Fig. 7A; Fig. S21). These abnormalities resemble the seam cell lineage defects caused by perturbed cytokinesis and loss of spindle assembly checkpoint (Tarailo-Graovac et al., 2010; Ding and Woollard, 2017). Simultaneous visualization of cell boundaries and nuclei using AJM-1::GFP and SCMp:GFP, respectively revealed the presence of multinucleated seam cells in *gvd-1A(kp86)* L4 larvae, which confirmed the cytokinesis failure (Fig. 7B). However, the presence of multiple seam cells suggests that the initial seam cell divisions were unaffected in these larvae. Thus, like in the other lineages (see above), *gvd-1* appears to be essential in the seam lineage only during late larval development.

Additionally, we examined the formation of tail structures called rays in males, as these structures arise from the seam cell lineage. During L2 and L3 stages, the three posteriormost seam cells in males execute a pattern of cell divisions that differs from their hermaphrodite counterparts and generate nine precursors of tail structures called rays, which are involved in the male mating behavior (Sulston and Horvitz, 1977; Lints and Hall, 2009). These ray precursors further divide between mid-L3 and mid-L4 stages and form nine rays by the late L4 stage. In contrast to wild-type males, *gvd-1A(kp86)* males did not produce any ray-like structures (Fig. 7C), which indicates a role for *gvd-1* in the seam cell lineage of males as well.

### Loss of *gvd-1A* function does not disrupt but delays the G1/S transition in P6.p

**Fig. 8.**
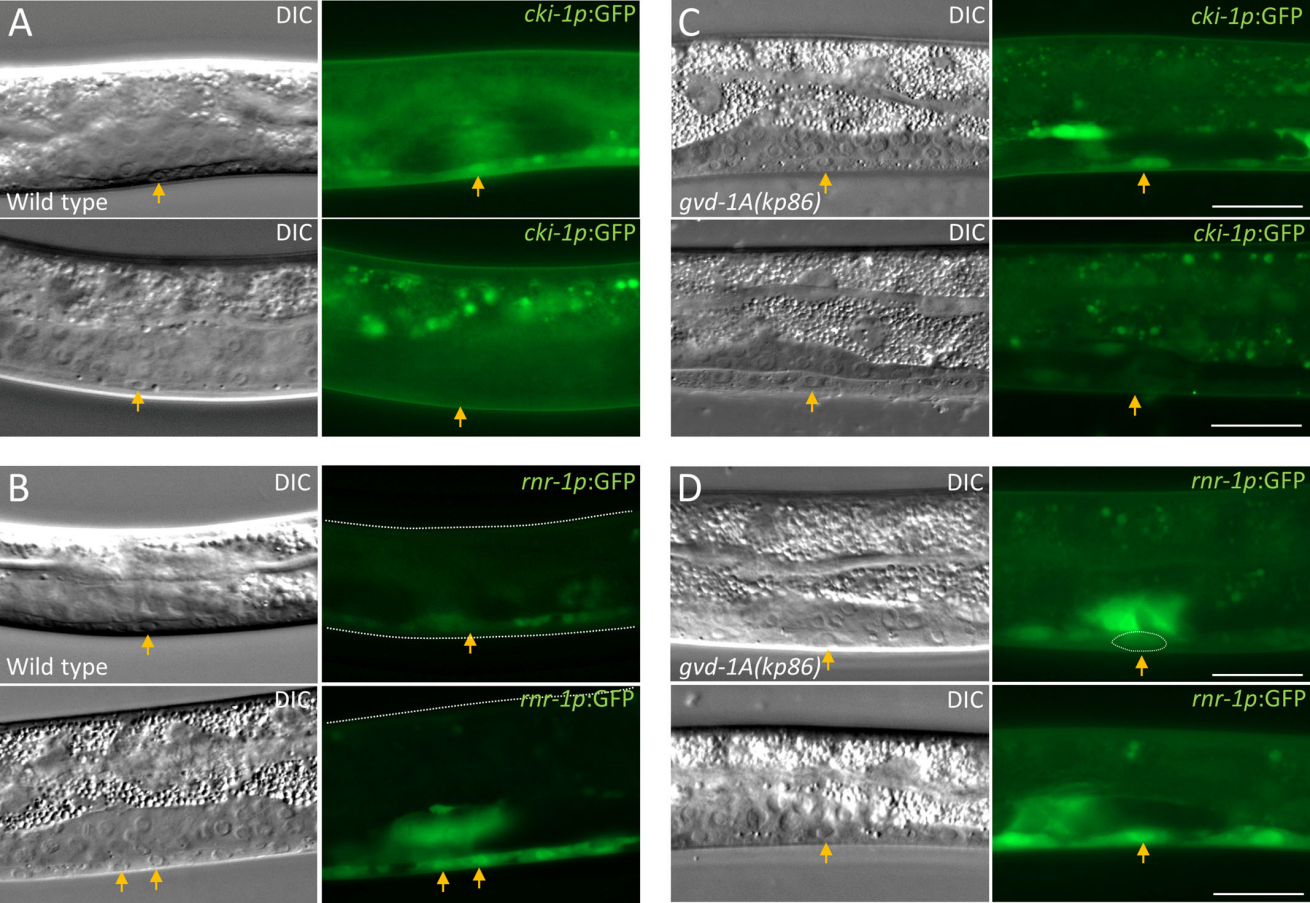
**Loss of *gvd-1A* function does not affect S phase entry in P6.p.** (A,C) In both wild-type and *gvd-1A(kp86)* larvae, the G1 phase marker *cki-1p*:GFP is present in P6.p (arrows) at 39 h (wild type) and 68 h [*gvd-1A(kp86)*] post-egg laying (top), and disappears before P6.p divides (bottom). (B,D) The S phase marker *rnr-1p:*GFP fluorescence is initially faint in both wild-type (39 h) and *gvd-1A(kp86)* (68 h) P6.p, but becomes brighter in the two descendants wild-type P6.p (bottom, left) and in *gvd-1A(kp86)* P6.p although it is yet to divide. Scale bar: 20 μm.

In L1 larvae, the cyclin-dependent kinase (CDK) inhibitor CKI-1 maintains blast cells such as P6.p in the G1 phase of the cell cycle. Consistently, expression of GFP driven by the ribonucleotide reductase-1 (*rnr-1*) promoter (*rnr-1p*:GFP), which serves as an S phase marker, is absent in P6.p at the L1 stage (Hong et al., 1998). Resumption of the cell cycle at the late L3 is marked by the disappearance of *cki-1* promoter driven GFP (*cki-1p*:GFP) and the appearance of *rnr-1p:GFP* which persists in P6.p descendants till the onset of terminal differentiation (Hong et al., 1998). We introduced these two reporters into the *gvd-1A(kp86)* strain and examined if their expression patterns in P6.p and its descendants switched normally during larval development. As shown in Fig. 8 and Fig. S22, in majority of *gvd-1A(kp86)* larvae, *cki-1p*:GFP disappeared and *rnr-1p*:GFP appeared in P6.p before it divided, indicating that the G1/S transition in P6.p is not dependent on *gvd-1*. However, the disappearance of *cki-1p:GFP* was substantially delayed in *gvd-1A(kp86)* larvae (Fig. S22), which points to a delayed G1/S transition or S phase entry, in the absence of GVD-1.

### *gvd-1* functions in both soma and germ line

**Fig. 9.**
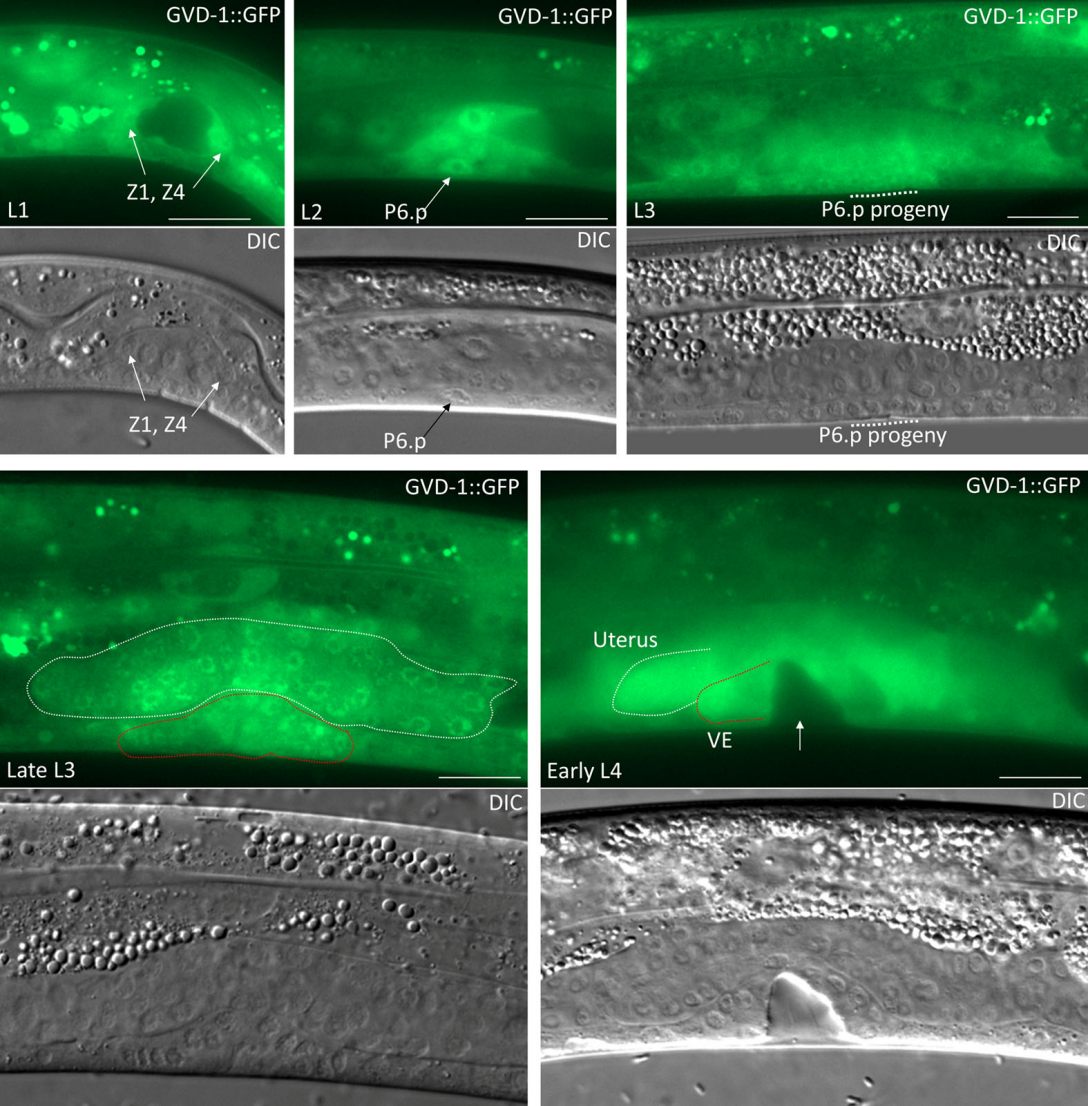
**Expression patterns of *gvd-1p*:GVD-1::GFP in the developing somatic gonad and vulva.** (Top) GVD-1::GFP is expressed in the somatic gonadal precursors Z1 and Z4 in L1 larva and in P6.p and its descendants in L2 and L3 larvae, respectively. (Bottom, left) GVD-1::GFP expression is seen in the developing uterus (white dotted lines) and vulva (red dotted lines) at the L3 stage, and persists in the uterus and vulval epithelium (VE) at the L4 stage (bottom, right). Scale bar: 20 μm.

**Fig. 10.**
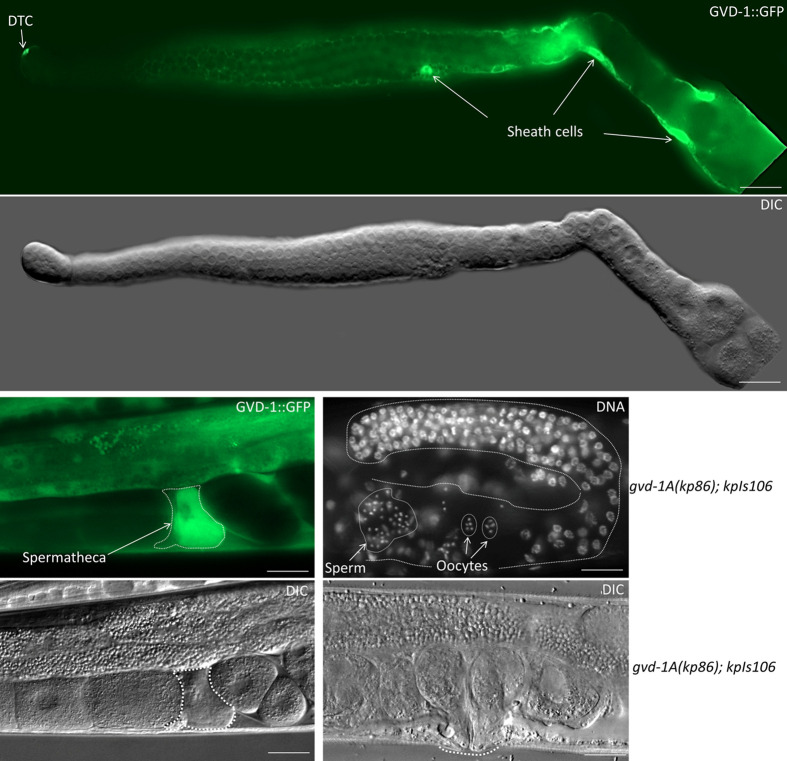
***gvd-1p*:GVD-1::GFP is expressed in the sheath cells and spermatheca in adults.** (Top) extruded germ line in which GVD-1::GFP fluorescence is clearly visible in the cell bodies (arrows) and the mesh-like cytoplasmic extensions of sheath cells, and in the DTC. (Bottom, left) a section of whole worm showing the expression of GVD-1::GFP in the spermatheca (white dotted line). Note that GVD-1::GFP is excluded from sperm (top and bottom left, and a dark area roughly in the middle of the outlined area). (Bottom, right) the *gvd-p:GVD-1::GFP* (*kpIs106)* transgene restores gametogenesis (top) and vulva development in *gvd-1A(kp86)* animals (compare with images in Fig. 2). Scale bar: 20 μm.

Next, we wished to determine the expression pattern of *gvd-1*. After initial unsuccessful attempts to tag the endogenous locus with GFP or the 3xFLAG epitope, we succeeded in generating a transgenic line using the Mos1-mediated single-copy insertion (MosSCI) method (Frokjaer-Jensen et al., 2012). The transgene, *kpIs106*, in this line contains 2.26 kb of genomic DNA immediately upstream of the *gvd-1* start codon, the coding sequences of GVD-1 followed by GFP, and 1.3 kb of genomic DNA downstream of the GVD-1 stop codon. Animals carrying *kpIs106* expressed GVD-1::GFP in various somatic tissues including the somatic gonad, vulva and seam cells (Fig. S23). Subcellularly, GVD-1::GFP prominently localized to the nucleus but could be observed in the cytoplasm as well. GVD-1::GFP expression in the somatic gonad started at the L1 stage and persisted until the adult stage; its expression was clearly visible in Z1 and Z4, their progeny and the SGP at the L1, L2 and early L3 stages, respectively. In the vulval lineage, GVD-1::GFP was present in P6.p and its descendants. In late L3 and early L4 larvae, we noticed GVD-1::GFP in the spermathecal/uterine precursors (Fig. 9). In adults, GVD-1::GFP could be observed in DTCs, Sh cells and spermatheca (Fig. 10). This transgene rescued the somatic gonadal and vulval defects of *gvd-1A(kp86)*, which indicates that the GFP fusion did not functionally compromise GVD-1, and that the observed expression pattern likely reflects that of the endogenous *gvd-1* locus. Possibly due to germline silencing, GVD-1::GFP was absent in the germ line; consistently, *kpIs106* did not rescue the germ cell proliferation defect of *gvd-1A(kp86)*. To circumvent the potential silencing of *kpIs106* in the germ line, we expressed GVD-1::GFP in *gvd-1A(kp86)* germ lines using the germline-specific *pie-1* promoter, and found that this transgene (*kpIs7*) was able to partially rescue the germ cell proliferation defect (Fig. S24). Since *kpIs7* expression was restricted to the germ line, the sheath cell defect would not have been rescued in these animals, which probably explains why the germ cell proliferation in the *gvd-1A(kp86); kpIs7* germ lines was not fully restored as sheath cells have been known to promote germ cell proliferation (McCarter et al., 1997; Killian and Hubbard, 2005). As expected, when introduced together, *kpIs106* and *kpIs7* rescued the somatic and germ line defects, and restored fertility in *gvd-1A(kp86)* animals, although there was a slight developmental delay and reduction in brood size when compared to wild type (Fig. S24). These observations support that *gvd-1* is expressed both in the soma and the germ line and indicate that the germline expression of *gvd-1* is essential for its ability to promote germ cell proliferation. Consistent with the involvement of somatic gonadal cells in oocyte maturation (McCarter et al., 1997; Hall et al., 1999), expression of GVD-1::GFP in the germ line alone was unable to restore fertility in *gvd-1A(kp86)* animals (Fig. S24).

## DISCUSSION

Results presented here show that the *C. elegans* gene *gvd-1* is essential for late larval development. Mutation in *gvd-1* affects development in several post embryonic cell lineages that neither share ancestry nor the functions of terminally differentiated descendants. A simple explanation for this is that GVD-1 functions in a housekeeping cellular process, and that the maternally produced GVD-1, which is sufficient for embryonic and early larval stages, decreases in late larvae below the threshold required for its activity. Our current results do not formally rule out this possibility. However, at least three of our observations are inconsistent with this notion. One, the amino acid sequence of GVD-1 is conserved only in the nematode lineage, which does not support a conserved housekeeping function. Two, *gvd-1* mutant larvae, although smaller than the wild type, exhibited normal locomotion and did not die prematurely. If GVD-1 functioned as a housekeeping protein, these larvae would have died prematurely when the maternal supply was exhausted. Three, depletion of GVD-1 by dsRNA injection into *gvd-1A(kp86/kp86)* animals carrying the rescuing transgenes phenocopied the *kp86* allele but did not cause embryonic or early larval defects. Therefore, we favor the model in which GVD-1 has a specific role that is critical for late larval development.

In each of the postembryonic lineages affected by *gvd-1A(kp86)*, the cell divisions were defective: in the germ line, germ cells failed to proliferate; in the vulval precursor P6.p, the first division was substantially delayed, and the second division perhaps did not occur; and cytokinesis failed in seam cells. These observations suggest a role for *gvd-1* in cell division. Consistently, we noticed that the *cki-1p*:GFP persisted longer in the *gvd-1A* mutant P6.p, which points to a delay in the G1/S transition. However, as indicated by the rescuing *GVD-1::GFP* transgenes, GVD-1 expression was not limited only to those blast cells that divide later, and *gvd-1A(kp86)* larvae exhibited an overall growth defect in addition to the postembryonic cell division defects. Thus, it is possible that GVD-1 may function in a process other than cell division.

The *kp20* allele of *gvd-1* reveals a genetic redundancy between *gvd-1* and *puf-8* in the germ line. PUF-8 has been shown to regulate the translation of several mRNAs in the germ line through 3′ UTR binding (Mainpal et al., 2011; Vaid et al., 2013; Maheshwari et al., 2016). Therefore, one explanation that may explain the redundancy is that both GVD-1 and PUF-8 influence the same target gene(s) in the same direction, but at different steps of gene expression. For instance, if GVD-1 is indeed present in the nucleus as indicated by our transgene reporters, it may regulate the transcription of mRNA(s) whose translation is regulated by PUF-8. Thus, it may be worthwhile to test if levels of any of the potential PUF-8's mRNA targets are affected in *gvd-1* mutant germ lines. Nevertheless, in the absence of knowledge on the kind of molecular activities that GVD-1 is capable of, other possible models deserve equal attention. It is also possible that in the altered translational landscape in *puf-8* mutant germ lines, even subtle perturbations cause severe defects. In any case, the observation that the *kp86* mutation causes severe germline defects on its own clearly shows that the genetic redundancy between *gvd-1* and *puf-8* in the germ line is limited.

## MATERIALS AND METHODS

### Growth and maintenance of *C. elegans* strains

The various *C. elegans* strains used in this study were maintained on lawns of the *E. coli* strain OP50 on agar plates containing nematode growth medium (NGM) as described (Brenner, 1974). Unless specified, the strains were maintained at 20°C. The various strains used are listed in Table S4. Strains with various allelic combinations, including the ones with cell-specific transgene reporters and rescuing *gvd-1::gfp* transgenes, were constructed through standard genetic crosses.

### RNA interference

Initial RNAi-mediated depletions performed during mapping of the *kp20* allele (Table S3 and Fig. S10) and the depletion of GLD-1 (Fig. S14) were carried out by bacterial feeding described by Timmons et al. with modifications (Timmons et al., 2001; Mainpal et al., 2011). The RNAi-mediated depletion of GVD-1 described in Fig. S20 was carried out by dsRNA injection. The dsRNA was prepared by annealing the sense and antisense RNAs, which were generated by *in vitro* transcription using T7 RNA polymerase (ThermoFisher Scientific). To generate the templates for *in vitro* transcription, the entire coding sequence of *gvd-1* was obtained by RT-PCR from total RNA using PCR primers KS4769 and KS4770 and cloned in the pSV2 plasmid vector (Mainpal et al., 2011). The insert from the resulting plasmid pAP9 was PCR-amplified using KS6417 and KS2484, and KS2483 and KS6418 primers and used as templates for the *in vitro* transcription of the sense and antisense strands, respectively. dsRNA was injected into only one gonad arm of young adults – obtained by selecting L4 larvae about 24 h prior to injection – at a concentration of 200 ng/μl in TE buffer (pH 7.0). Injected animals were allowed to recover on OP50 lawns for about 24 h and were shifted to fresh lawns. Embryos laid on the fresh lawns for about 12 h were used for examining the phenotype. The sequences of all oligonucleotides used are listed in Table S5.

### Genetic mapping of *kp20*

Standard two-factor genetic crosses, as illustrated in Fig. S1, were performed using the mapping strains EG1000 and EG1020 to identify the chromosome on which the locus identified by *kp20* is located. In these crosses, *kp20* appeared to independently assort with all six marker mutations (Table S1). However, while generating a strain with the genotype *puf-8(zh17) rol-6(e187) / mnC1 k20 / kp20* using *puf-8(ok302) unc-4(e120) / mnC1 k20 / kp20* and *puf-8(zh17) rol-6(e187) / mnC1*, we noticed that *kp20* cosegregated with *puf-8(ok302)* and *unc-4(e120)* suggesting that *kp20* might be on the same chromosome as *puf-8* and *unc-4*, which is chromosome II. Quantitative analysis of the segregation pattern indicated that *kp20* indeed was present on chromosome II (Fig. S2 and Table S2).

We performed SNP-mapping to define a specific genetic interval in which *kp20* is located on chromosome II by following the mapping protocols described earlier with modifications (Doitsidou et al., 2010) (Fig. S3). In the scheme outlined in Fig. S3, since the fertile and sterile ‘unc’ worms were expected to be homozygous for *puf-8(ok302)* and *unc-4(e120)*, they both were expected to show linkage with N2-specific SNPs near the center of chromosome II where *puf-8* and *unc-4* are present. In addition, since *kp20* is on chromosome II, the N2-specific SNPs that show association with the sterile ‘unc’, but not with the fertile ‘unc’ phenotype, would indicate the presence of *kp20* in their vicinity. Initial chromosome mapping SNP analysis (Fig. S3) confirmed that *kp20* was on chromosome II, and further refined the genetic interval to −14 and −18. We analyzed the cosegregation of SNPs at genetic positions −18, −14 and −6 on chromosome II with *kp20*, and found that four out of five sterile ‘unc’ worms were homozygous for the N2 SNP at position −14, whereas the corresponding numbers were two out of five for both positions −18 and −6, which indicated that *kp20* was nearer to the map position −14 than to −6 or −18 on chromosome II.

Genome sequencing (carried out by the Genome Technology Access Center, Washington University, St. Louis, MO, USA) identified non-synonymous mutations in 11 genes between −18 and −6 on chromosome II in *puf-8(zh17) rol-6(e187)/mnC1 k20/kp20* animals (Table S3). Of these, RNAi-mediated depletion of only W10D9.6 (map position −15.6) resulted in *puf-8(ok302)*-dependent sterility (Table S3), suggesting that *kp20* is an allele of *W10D9.6*. We confirmed the G-to-A substitution in *W10D9.6* revealed by the whole-genome sequencing by sequencing the *W10D9.6* locus in *puf-8(ok302) unc-4(e120) / mnC1 k20/kp20* animals (Fig. S4). Additionally, the transgene *kpIs7*, which expresses W10D9.6::GFP in the germ line, restored fertility in 95% of *puf-8(ok302) kp20* animals (*n*=150).

### Generation of the *kp86* and *kp91* alleles

The putative null alleles of *gvd-1* A and its potential paralog *W10D9.3*, *kp86* and *kp91* respectively, were generated using the CRISPR/Cas9 protocol described by Arribere et al. (Arribere et al., 2014). The sgRNA expression plasmids were generated as described earlier (Kumar and Subramaniam, 2018). The plasmid pAP21 was used to express the sgRNA, and the oligonucleotide KS4988 was used as the repair template for generating the *kp86* mutation. pAP21 was generated by annealing oligonucleotides KS4996 and KS4997 and inserting at the *Bsa*I site of pAP20 (Kumar and Subramaniam, 2018). For engineering *kp91* mutation, two sgRNA vectors, pKS232, which contains the annealed KS6468 and KS6469, and pKS233, which contains the annealed KS4670 and KS4671, were used along with the KS6473 oligonucleotide as the repair template. The concentrations used were 20 ng/μl for sgRNA plasmids and 1 μM for repair templates. The progeny of injected animals expressing the marker-edit phenotype were cloned and allowed to lay embryos for 2 days, following which they were screened for the presence of desired mutations by single-worm PCR using primers KS5071 and KS5073, which were specific for the *kp86* mutation, or KS6474 and KS6475, which were specific for the *kp91* mutation.

### Generation of transgenic lines

The transgenic line IT1074, which expresses GVD-1::GFP in the germ line, was generated through biolistic bombardment method described by Praitis et al. with modifications (Praitis et al., 2001; Jadhav et al., 2008). The plasmid pAP16, which drives GVD-1::GFP under control of *pie-1* promoter and 3′ UTR, was constructed by modifying the pKS114 vector described earlier (Mainpal et al., 2011). First, the coding sequences of H2B were removed by digestion with *Spe*I and *Nar*I, end-filled and religated. Second, the coding sequence of *gvd-1* was PCR-amplified using primers KS4780 and KS4781 and inserted at the *Bam*HI site upstream of GFP coding sequences.

The transgenic line IT1244, which expresses GVD-1::GFP under the control of *gvd-1* promoter and 3′ UTR, was generated using Mos1-meidated single copy insertion (MosSCI) method (Frokjaer-Jensen et al., 2012). The transgenic plasmid pAP51 was constructed as follows. A 3.8 kb genomic DNA fragment, which starts at 1.6 kb upstream of the *gvd-*1 start codon and ends at the codon for the last amino acid, was PCR-amplified from *C. elegans* genomic DNA using primers KS4921 and KS4781 and cloned in pSV2 using the TA cloning method. In the resulting plasmid, a DNA fragment containing the coding sequences of GFP, which was PCR-amplified from pKS114 using primers KS1468 and KS3324, was inserted between *Spe*I and *Apa*I sites. In the resulting plasmid, a 1.6-kb genomic fragment immediately downstream of the *gvd-1* stop codon was PCR-amplified from genomic DNA using primers KS4924 and KS4940 and inserted at the *Apa*I site. The resulting plasmid was cut with *Bgl*II, to release a 6.0-kb fragment, which contains *gvd-1* promoter, coding sequences of GVD-1 and GFP, and the *gvd-1* 3′ UTR. This fragment was inserted at the *Bgl*II site of the MosSCI vector pCFJ356 to generate pAP51. The transgene was inserted at the *cxTi10816* locus on chromosome IV using the strain EG6703 (Frokjaer-Jensen et al., 2012).

### Fluorescence microscopy

The staining of germ lines and whole animals with DAPI was carried out as described in Vishnupriya et al., 2020. For immunostaining with anti-phosphohistone H3 antibodies (anti-PH3; Sigma, catalog number H0412), extruded germ lines were fixed in formaldehyde fixative [2% formaldehyde, phosphate-buffered saline (PBS), 0.8 mM EGTA, 1.6 mM MgSO_4_] for 2 min at room temperature, followed by incubation in pre-chilled methanol at −20°C for 15 min and in pre-chilled acetone at −20°C for 10 min. The fixed germ lines were washed thrice and incubated for 30 min in PBT (PBS / 0.1% Triton-X-100 / 0.1% bovine serum albumin), followed by incubation in the primary antibody, which was diluted 1:1000 with PBT, at 4°C for 15 h with gentle rocking. The germ lines were then washed thrice with PBT and incubated in the secondary antibody (Jackson ImmunoResearch, 711-545-152), which was diluted 1:500 in PBT for 4 h at room temperature. Subsequent washing and mounting were performed as described earlier (Vishnupriya et al., 2020).

Fluorescence signals from fixed germ lines (DAPI and anti-PH3), and fluorescence reporters in live whole animals and extruded germ lines were observed using a Zeiss Axio Imager M2 fluorescence microscope and imaged using a Zeiss Axiocam 506 Mono CCD camera. Images were processed using Adobe Photoshop CS5; exposure conditions and processing were identical for all images presented in the same panel.

## Supplementary Material

10.1242/biolopen.059978_sup1 Supplementary information
