# Supplementary material for: The C. elegans gene gvd-1 promotes late larval development and germ cell proliferation
Source: Biol Open. 2023 Jun 30;12(7):bio059978. doi: 10.1242/bio.059978 (PMC10320718; doi:10.1242/bio.059978)
Supplement: Supplementary information [file biolopen-12-059978-s1.pdf]

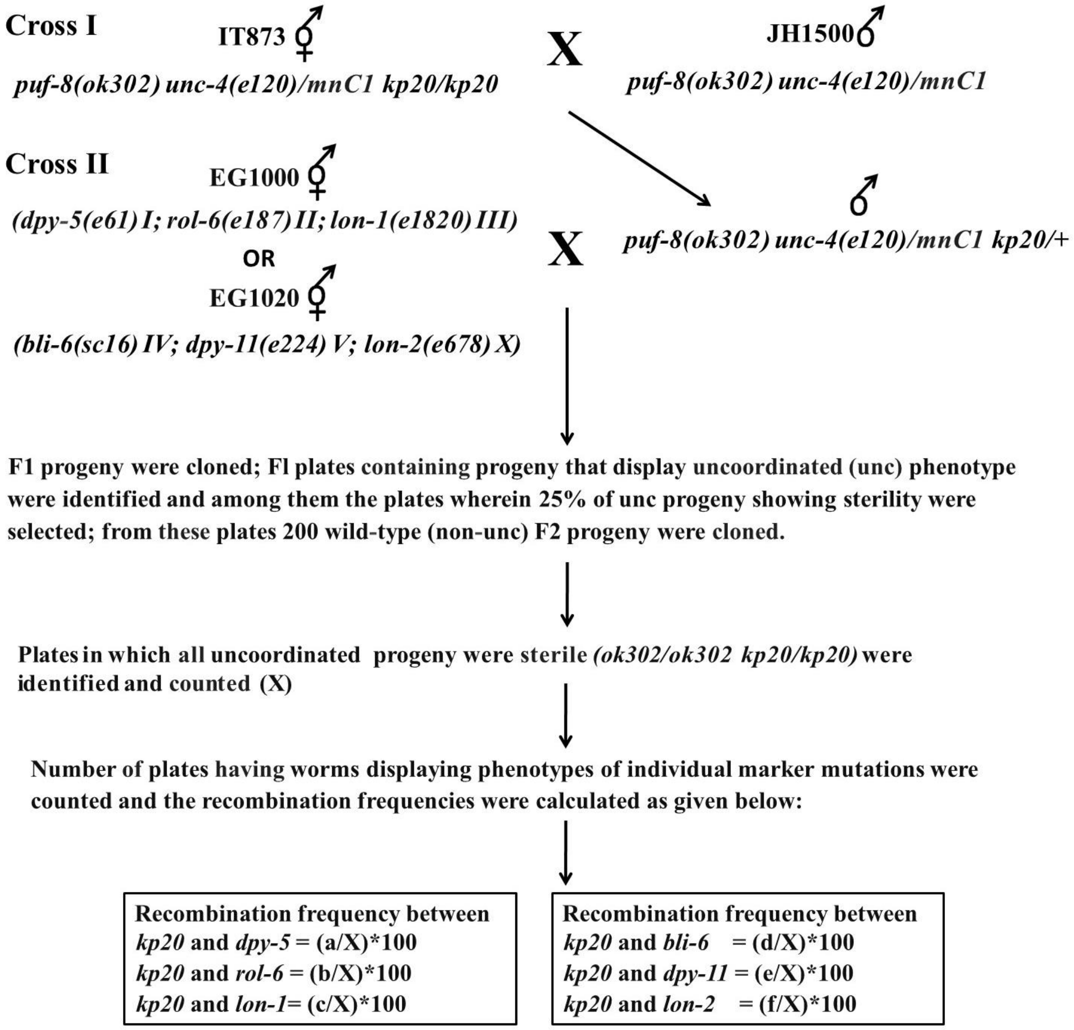

Fig. S1. Schematic representation of the two-factor mapping crosses

Biology Open • Supplementary information

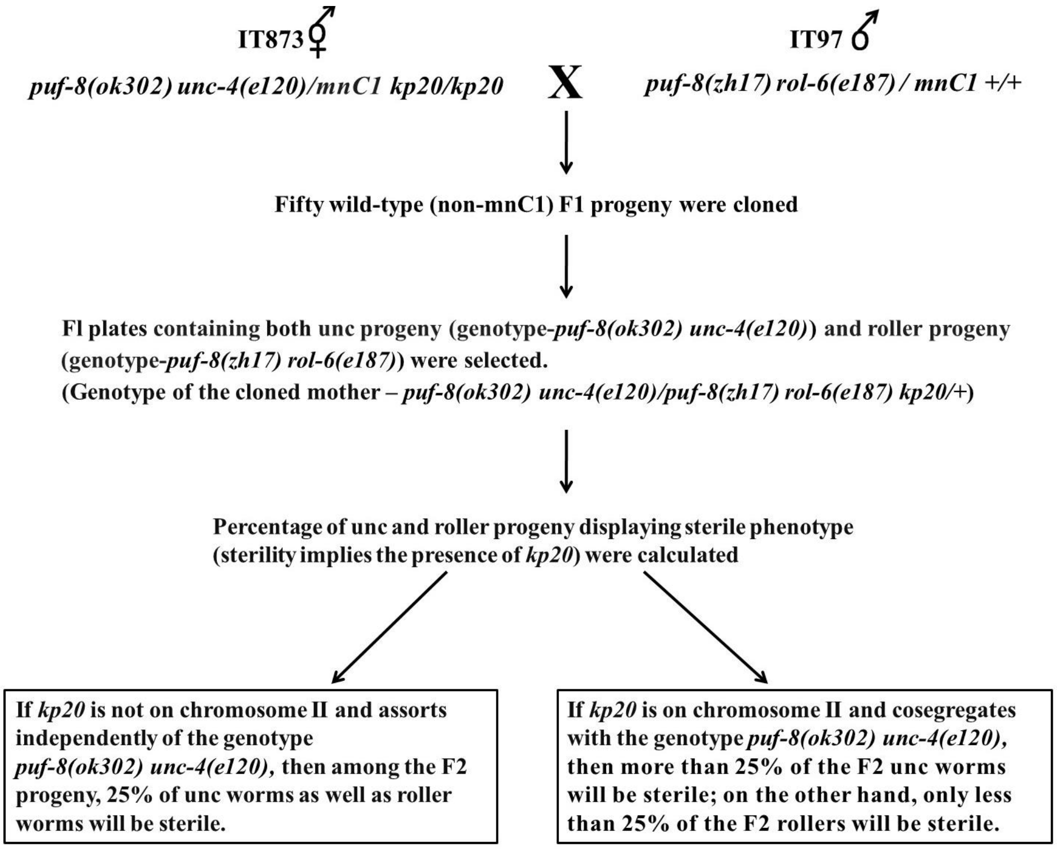

Fig. S2. Schematic representation of the segregation analysis of *kp20* with *unc-4(e120)* *and rol-6(e187)*

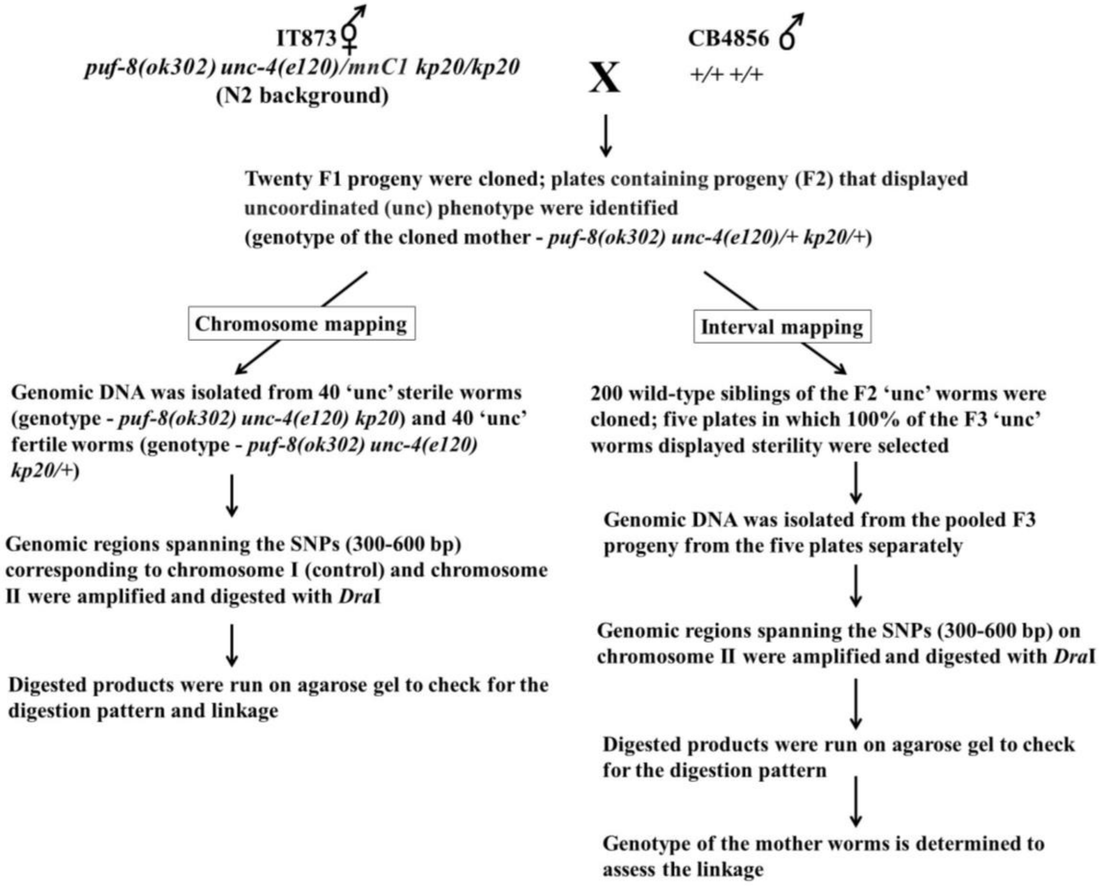

Fig. S3. Schematic representation of the SNP crosses

Biology Open • Supplementary information

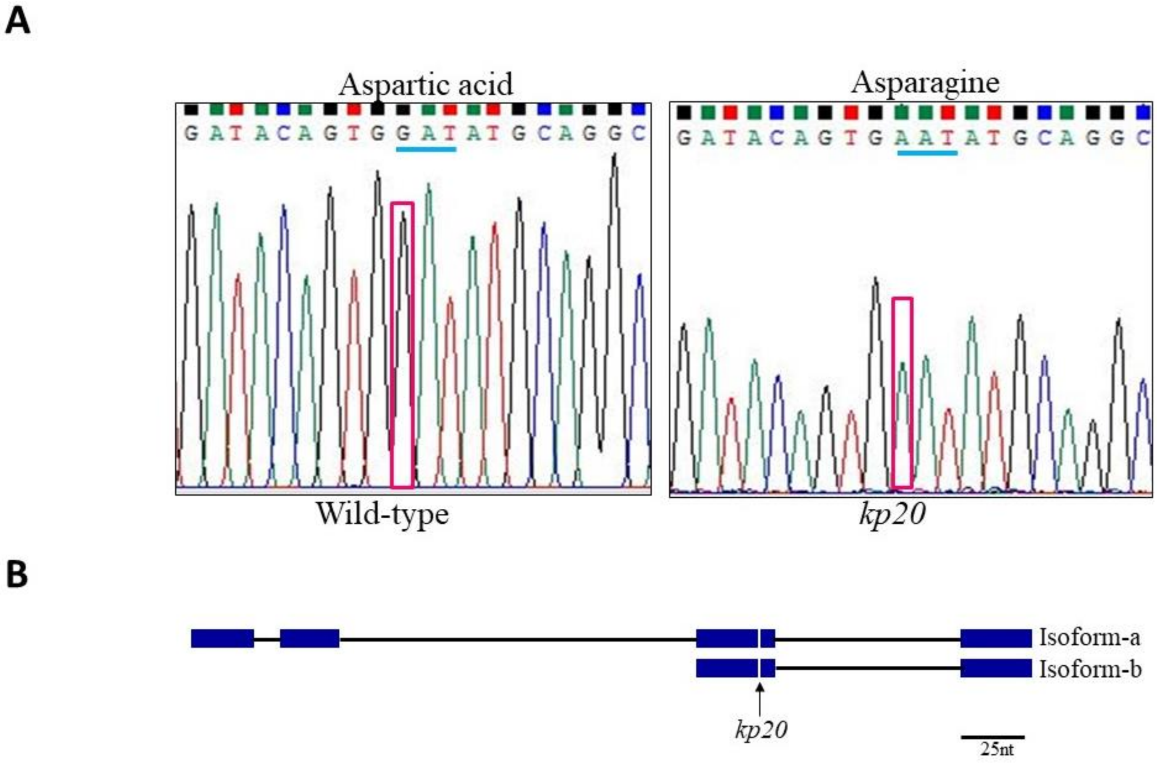

Fig. S4. ***kp20* is a missense mutation that substitutes aspartic acid-124 with asparagine.** (A) Electropherograms showing the G→A substitution that changes the GAT codon for aspartic acid to the AAT codon for asparagine. (B) Schematics showing the exons (blue bars) and introns (black lines) and their relative positions in the two isoforms of *gvd-1*. The drawing and the scale bar are based on the data available at www.wormbase.org for W10D9.6.

Biology Open • Supplementary information

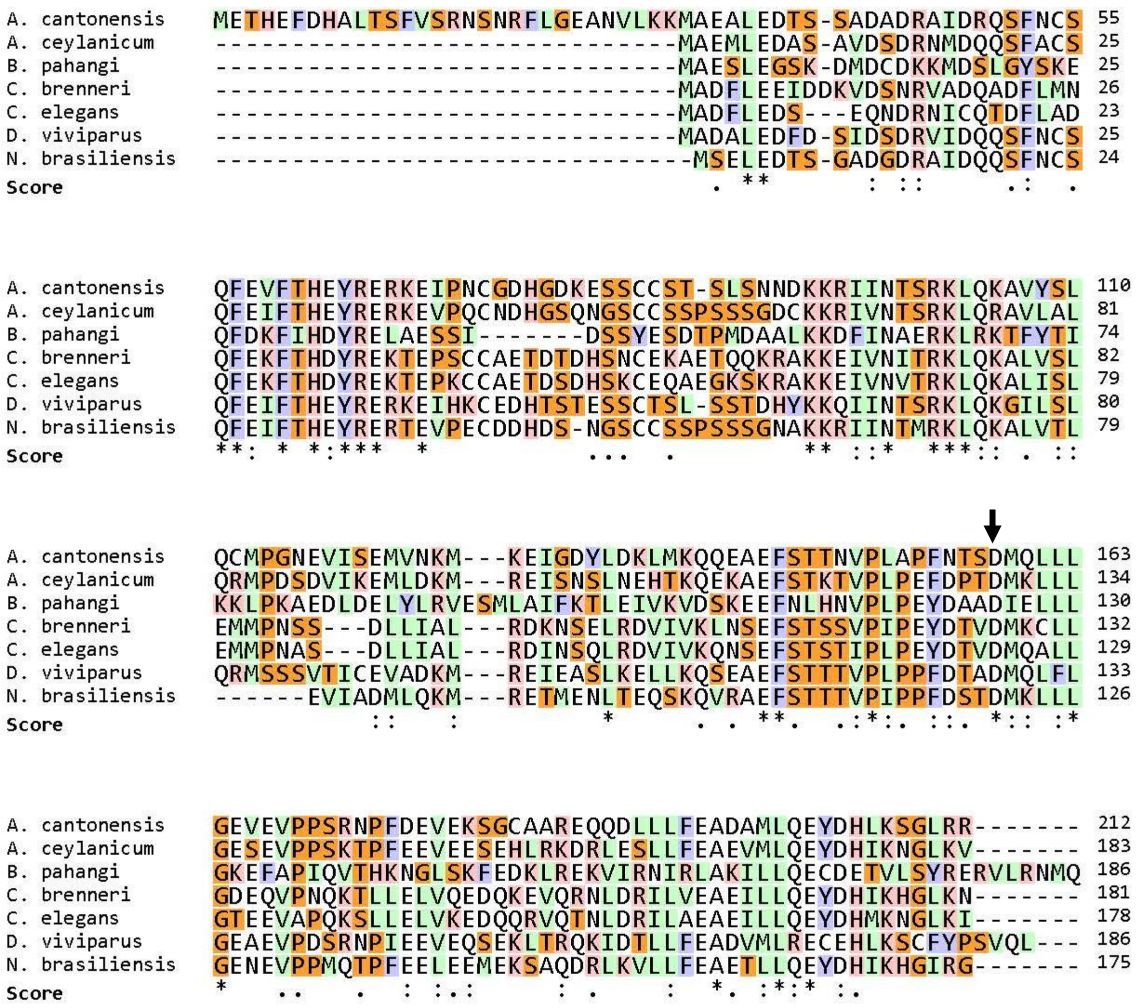

Fig. S5. Alignment of the amino acid sequences of GVD-1A nematode orthologs. Amino acid sequences of the indicated species have been aligned using the CLUSTALW program supplied with the DNA DYNAMO software package. GenBank accession numbers: *Angiostrongylus cantonensis* - KAE9420333.1; *Ancylostoma ceylanicum* - EPB73907.1; *Brugia pahangi* - VDN91815.1; *Caenorhabditis brenneri* - EGT52711.1; *Caenorhabditis elegans* - NP_001254012.1; *Dictyocaulus viviparus* - KJH45316.1; and *Nippostrongylus brasiliensis* - VDL62758.1. The aspartic acid affected by *kp20* (arrow) is conserved in all seven species. Identical amino acids are indicated by an asterisk (*), and the ones with very similar side chains and somewhat similar sides chains are indicated by two dots (:) and by single dot (.), respectively.

Biology Open • Supplementary information

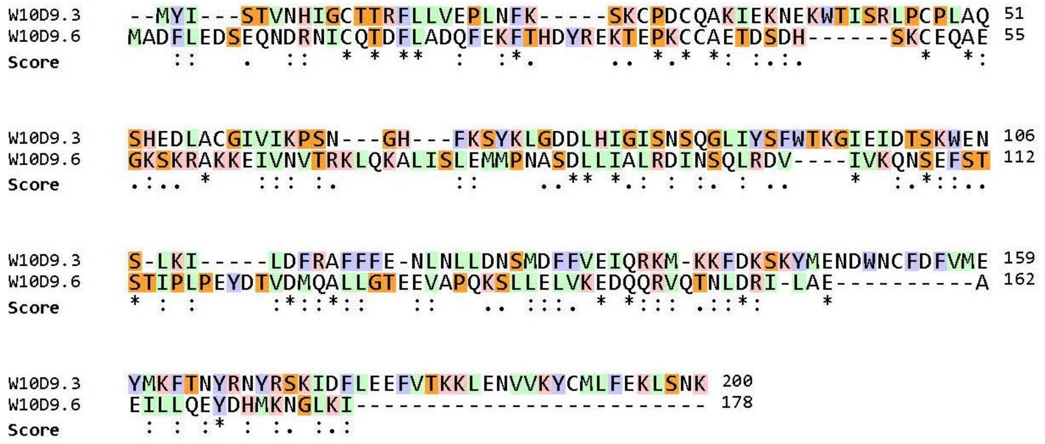

Fig. S6. Alignment of the amino acid sequences of GVD-1A (W10D9.6) and its potential paralog W10D9.3. Arrow points to the aspartic acid affected by *kp20*; Scores are the same as in Fig. S5 legend.

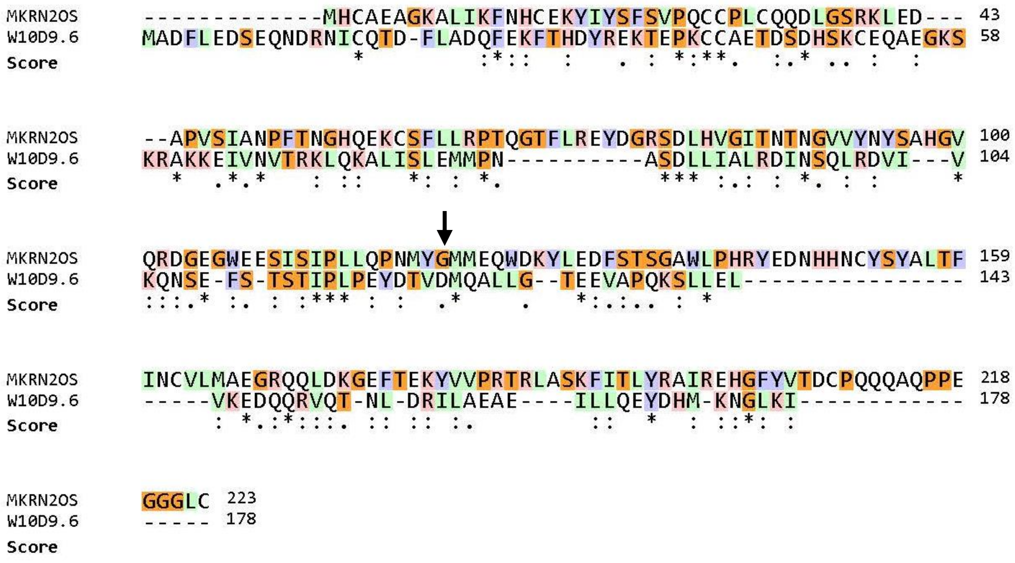

Fig. S7. Alignment of the amino acid sequences of GVD-1A (W10D9.6) and its potential human ortholog MKRN2OS. Arrow points to the aspartic acid in GVD-1 affected by *kp20*; Scores are the same as in Fig. S5 legend. Accession number for MKRN2OS is NP_001182208.1.

Biology Open • Supplementary information

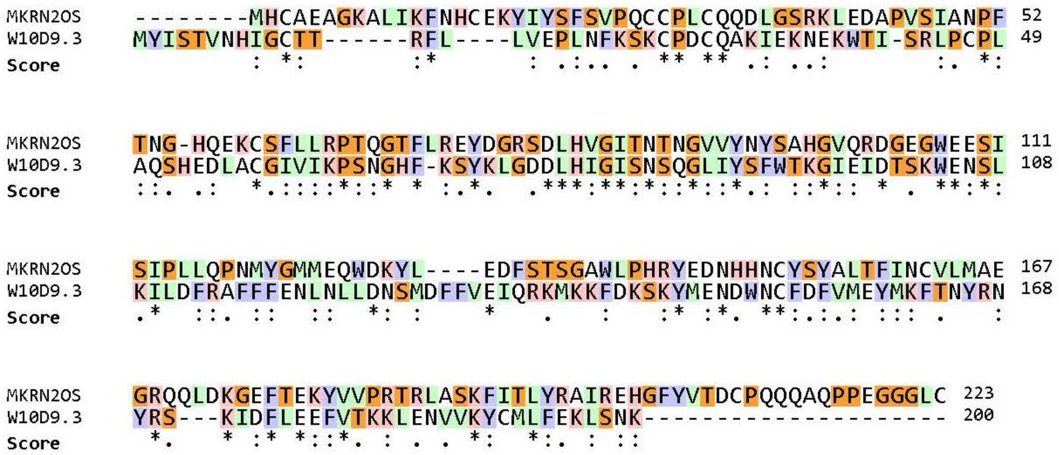

Fig. S8. Alignment of the amino acid sequences of W10D9.3 and its potential human ortholog MKRN2OS. Scores are the same as in Fig. S5 legend.

Biology Open • Supplementary information

```
W10D9.3 intron-2  GTAGAAATCGAAATTCAGCAATTTCCAGTCAAAATTATACATTTTCAATGCA 52
W10D9.6 intron-2  ----GTATGGAA-AAATGGGAAAAATATCGATTTTCAGCATTTTGCACATAA 47
Score                   ** ***     *  *     *   *   * *    *** **    *

W10D9.3 intron-2  AAATTCGAGTTCAGCGCCTCAAAATACAGCTATAACAAATTGTGTGGGGTTT 104
W10D9.6 intron-2  AAATTCGAATTCAGCCCCTCAATTTACCCCGTGGGAC-ACTTTACCTAATCC 98
Score             ******** ****** ******  ***  *        * * *      *

W10D9.3 intron-2  CTAACTCAAAAATTCAAAATTTTGGGTTACTGTAGCCCCAGAAGTACGCAAA 156
W10D9.6 intron-2  CATAGCTCAATAATTGAAGTTTTGGGCTACTGTAGCTCCAGGAGTACGCAAA 150
Score             *  *    ** * *  ** ******* ********* **** **********

W10D9.3 intron-2  CACCAACTACAGTACCCCAAAACACCTGATTTTACGACATTTTGCGAATTTC 208
W10D9.6 intron-2  CACCGACTACAGTACCCCAAAATATCTGATTTTACGACAT-TGGCGAATTTC 201
Score             **** ***************** * *************** * *********

W10D9.3 intron-2  GAGCTGAAAATTGATTTCACGGGGTAAAATGGGGTGCTGAATTAAAAAATCG 260
W10D9.6 intron-2  GAGCTGAAAATTGATTTCACGAGGTAAAATGACGTGCTGAATTCGAAAATTG 253
Score             ********************* *********  **********  ***** *

W10D9.3 intron-2  TTTTGAAAATTTCAGAAAAAATTTTTGAAAGTCTGAAATGTTGAGATTTTCA 312
W10D9.6 intron-2  TATTGAAAGTTTTTGGAAATGGCTTTGAAAGTGTGAAATTTG-AGAATTTTC 304
Score             * ****** ***  * ***    ********* ****** *  *** ***

W10D9.3 intron-2  GCCAAAATTCCGGATTTTGACCTTTTCCACTGAGAATTTCAAATTTCTAATG 364
W10D9.6 intron-2  GACAAAATTTCGGATTTTGACATTTTACACTGAGAATTTCAAATTTCTAATC 356
Score             * ******* *********** **** ************************

W10D9.3 intron-2  TAAAATTTGAATTCAGCGGCACAATTAACCCCATGAAAGACTTTTTTCCAGT 416
W10D9.6 intron-2  TAAAATTCGAATTCAGCGCCTCAATTTACCCACTATGACATTTTTGTCTGGT 408
Score             ******* ********** * ***** ****  *   * * **** **  **

W10D9.3 intron-2  CTGGTAGCACAAAAATTGAAGTTTTGGGTTACTGTAGCACCAAGAGTACGCA 468
W10D9.6 intron-2  TCCGTAGCTCAAAAATGGAAGTTTTGGGTTACTGTAGCTTCAAAAGTCCGCA 460
Score                ***** ******* *********************  *** *** ****

W10D9.3 intron-2  AACACCGACTACAGTACCCCAAAACACTTGATTTCACGACACTGGTGAATTT 520
W10D9.6 intron-2  AACACTAACTAAAGTACCCCAAAACACCCGATTTTACGACATTGACGAATTT 512
Score             *****  **** ***************  ***** ****** **  ******

W10D9.3 intron-2  CAAGCTGAAAATTGATGTGACGTGGTAAAATAGGATGGTGAATTCGAAAATC 572
W10D9.6 intron-2  TGAGCTGAAATTTGGCTTATAGGGGTAAAATGTGGTGCTGAATTCGAAAATC 564
Score               ******** ***   *   * ********  * ** **************

W10D9.3 intron-2  GTATTGAAAATTTCAGAAAATTTTTTTGAAATTTTTTTTTTTTGAAATTTTA 624
W10D9.6 intron-2  GTATTGAAAATTTTTGGAAATAATTTTGAAAGTCTAACATTTTGAGATTTTC 616
Score             *************  * ****  ******** * *    ****** *****

W10D9.3 intron-2  GGATTTTCAGGAAAAAAAAATTTTTGAAATTTTTTTTTCAACATAAAATC-- 674
W10D9.6 intron-2  GTACAATTTTATGATTTTTTCA--AAAAATTTTTATTTACATTCTTCTACTT 666
Score             * *   *      *            ******** ***  *        *

W10D9.3 intron-2  -TCAGGCTAAATCAAATTTTATCAAGGTTTTAAGACTCCCGAAGAGCAAATT 725
W10D9.6 intron-2  TTCCAGCCGGAAATTTTTTTAGAAAAATATTTCCAG---------------- 702
Score              **  **   *     *****  **  * **   *

W10D9.3 intron-2  TTCATCTAAAAAGTCTGAAAATAAATTAATTTTGCAAAAAAAAAAAATTTTT 777
W10D9.6 intron-2  ---------------------------------------------------- 702
Score

W10D9.3 intron-2  CAAATTTTATTCGAAATTTTGAAAAAATCTGAAATTTCAG 817
W10D9.6 intron-2  ---------------------------------------- 702
```

Fig. S9. Alignment of the nucleotide sequences of intron- 2 of *gvd-1A* (*W10D9.6*) and its potential paralog *W10D9.3*). Conserved residues are indicated by asterisk (*).

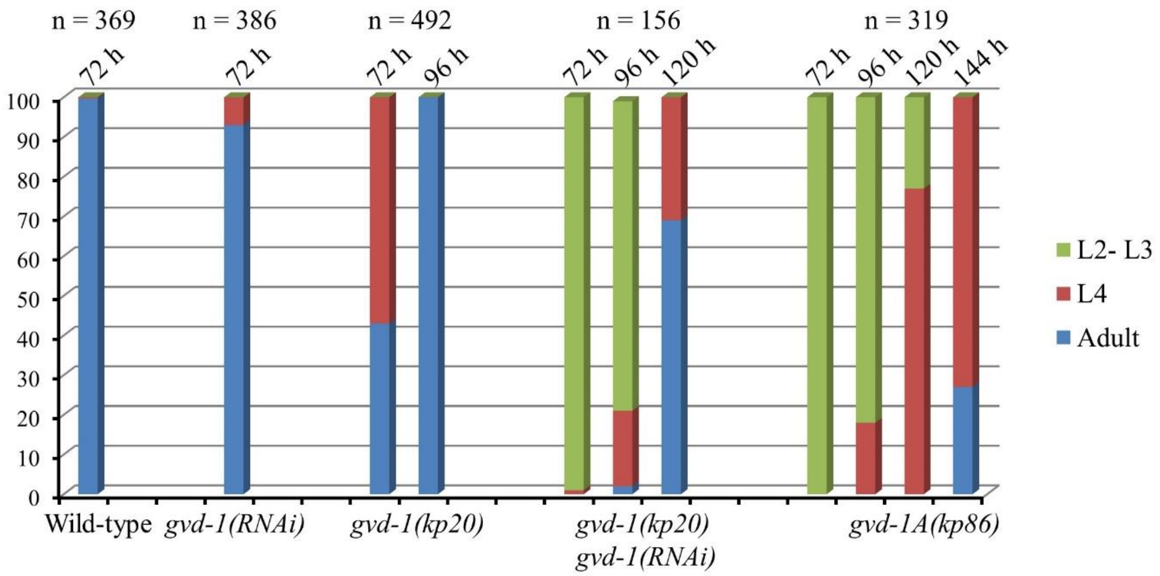

Fig. S10. *kp20* is a reduction-of-function allele of *gvd-1*. Each stacked bar shows the percentage of animals that attained the developmental stages indicated on the right at time points indicated above. The number of animals counted: wildtype = 369, *gvd-1(RNAi)* = 386, *gvd-1(kp20)* = 492, *gvd-1(kp20) gvd-1(RNAi)* = 156 and *gvd-1A(kp86)* = 319. h = number of hours post-egg laying. Appearance of vulval invagination, and the everted vulva (wild-type) or protruding vulva [*gvd-1A(kp86)*] were considered to mark the L4 and adult stages, respectively (see Fig. S18). Larvae at stages prior to vulval invagination were considered to be L2-L3.

Biology Open • Supplementary information

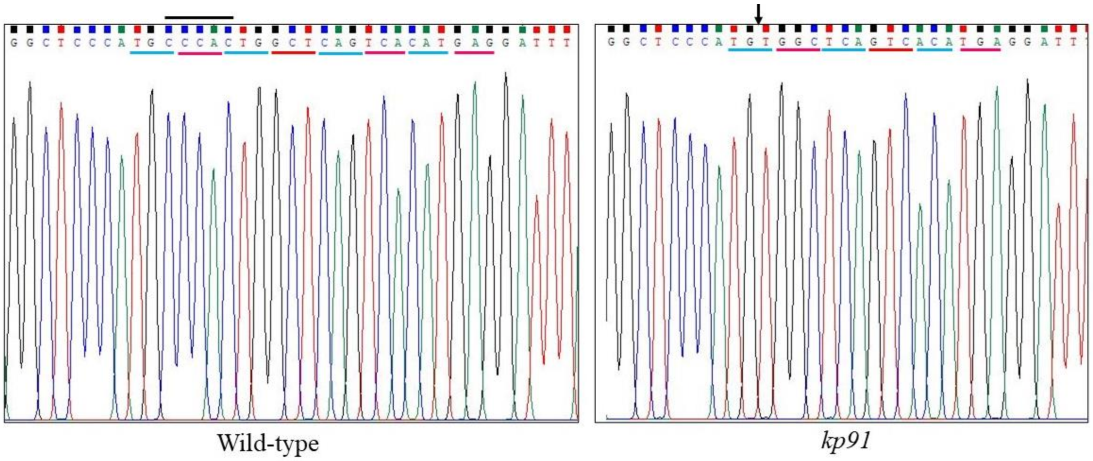

Fig. S11. Electropherograms of showing the nucleotides deleted in the *kp91* allele of *W10D9.3*. The *kp91* mutation deletes five nucleotides (marked by a black bar above in the wild-type sequence, and an arrow in the *kp91* sequence) and causes frameshift leading to premature termination. The wild-type and shifted reading frames are indicated by colored underlines.

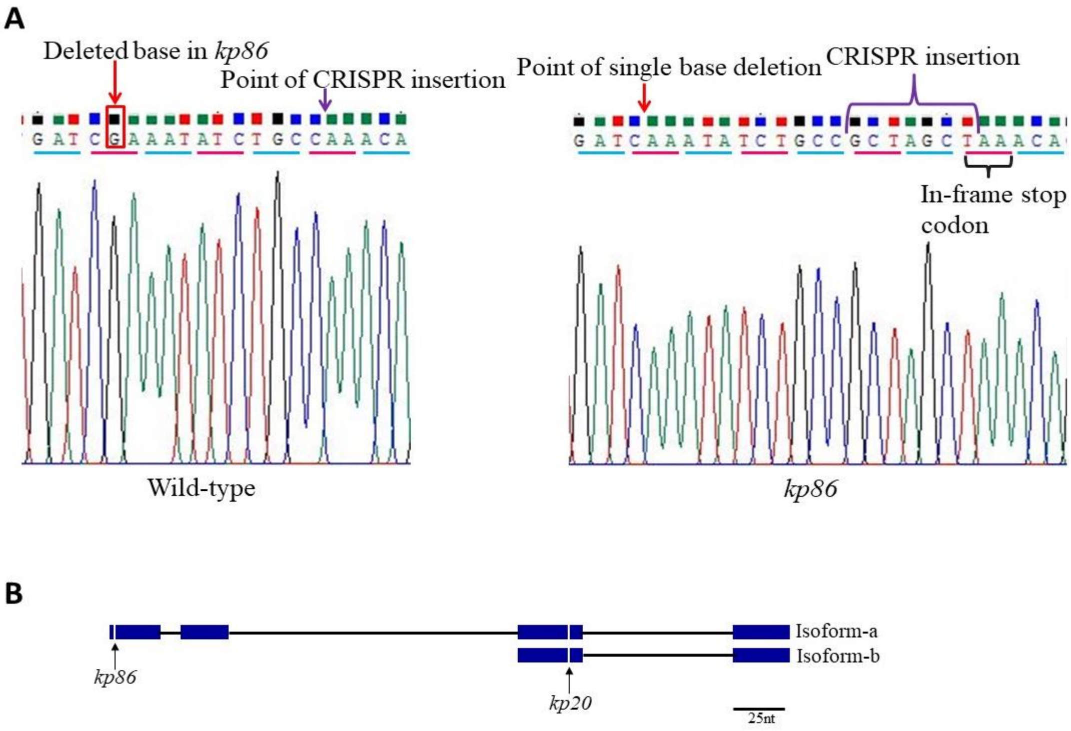

Fig. S12. Electropherograms comparing the sequences of wild-type and *kp86* alleles of *gvd-1*. (A) The wild-type and shifted reading frames are indicated by colored underlines. (B) Same bar diagrams as in Fig. S4B but shows the relative positions of the *kp20* and *kp86* mutations.

Biology Open • Supplementary information

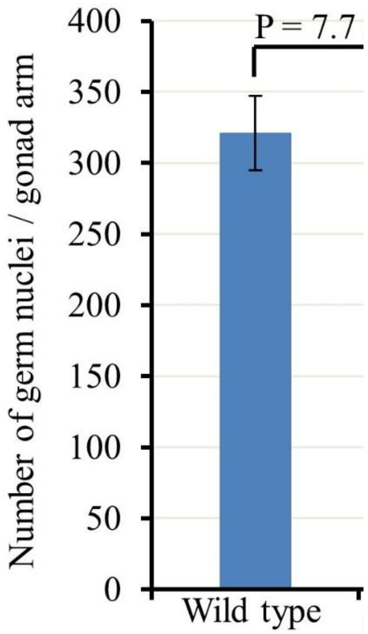

Fig. S13. ***gvd-1A(kp86)* germ line has fewer germ cells than the wild type**. Wild-type and *gvd-1A(kp86)* larvae were dissected at 62 hours and 72 hours post-egg laying, respectively. The germ cell nuclei in the extruded gonads were visualized by staining with DAPI and were counted using a fluorescence microscope. The values are average of the number of germ cell nuclei per gonad arm [n = 12 animals for wild type and 15 animals for *gvd-1A(kp86)*]. Error bars represent standard deviations, and the *P* values were calculated by two-tailed Student's *t*-test.

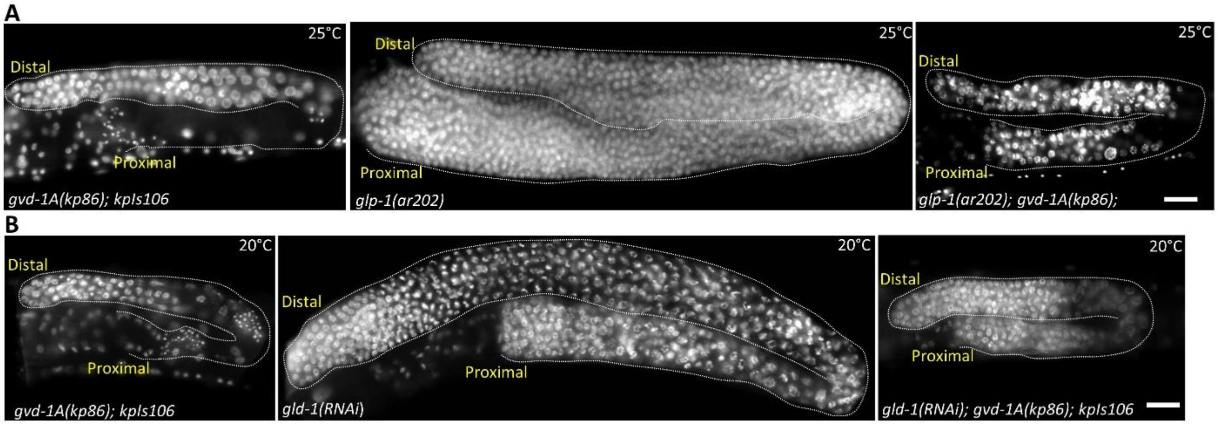

Fig. S14. ***gvd-1A(kp86)* suppresses germ cell tumors that form in *glp-1(ar202)* and *gld-1(RNAi)* animals.** Dissected germ lines of the indicated genotypes have been stained with DAPI. Note: The massive proliferation of germ cells observed in *glp-1(ar202)* and *gld-1(RNAi)* have been substantially reduced by the loss of *gvd-1A* function. Scale bar: 20 µm.

Biology Open • Supplementary information

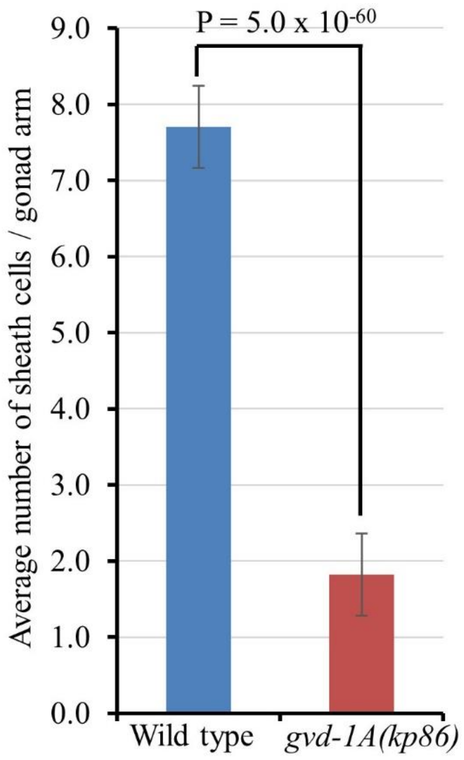

Fig. S15. ***gvd-1A*** **is required for the cell divisions in the sheath cell lineage.** The number of sheath cells expressing *lim-7p::*GFP, which marks the sheath cells, were counted in wild-type and *gvd-1A(kp86)* adults and plotted in this bar graph. The total number of gonad arms counted were 27 and 56, respectively for wild type and *gvd-1A(kp86)*. Error bars represent standard deviations, and the *P* values were calculated by two-tailed Student's *t*-test.

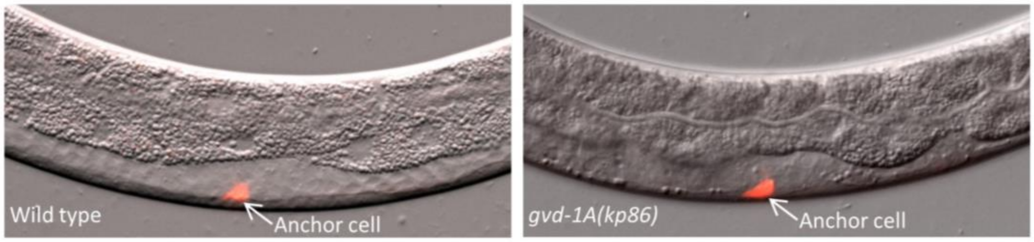

Fig. S16. Anchor cell formation does not depend on ***gvd-1A*****.** Ventral midbody region of L3-stage wild-type and *gvd-1A(kp86)* larvae are shown. In both, the anchor cell has been visualized using the qyls17 transgene, which expresses the anchor cell marker *zmp-1p*:mCherry (Schindler and Sherwood 2011). Merged fluorescence (*zmp- 1p*:mCherry) and differential interference contrast (DIC) images are shown.

Biology Open • Supplementary information

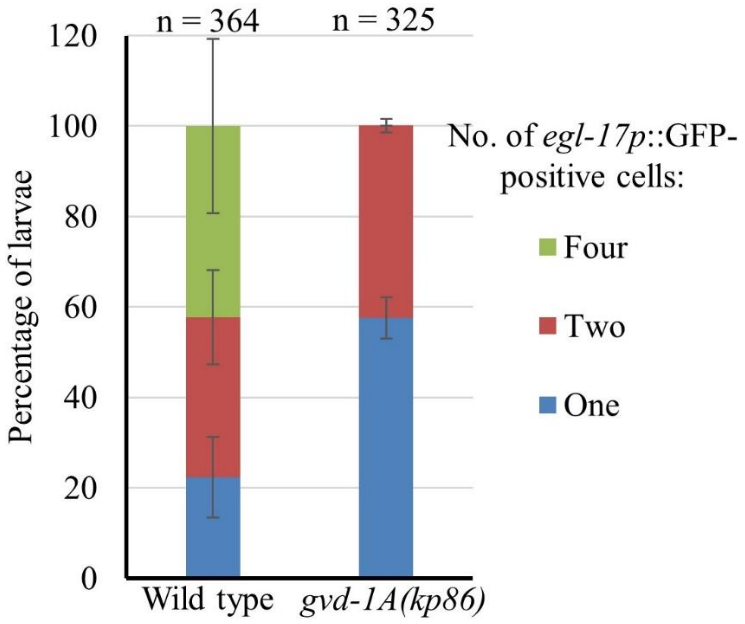

Fig. S17. The vulval precursor cell P6.p does not divide normally in the *gvd-1A(kp86)* mutant. The number of cells expressing *egl-17p::*GFP, which marks P6.p and its descendants, were counted in wild-type and *gvd-1A(kp86)* larvae at 48 hours and 70 hours post-egg laying [the earliest timepoint at which *kp86* homozygotes could be confidently identified], respectively, and plotted in this bar graph. The total number of larvae counted were 364 and 325, respectively for wild type and *gvd-1A(kp86)*. Error bars represent standard deviations for three replicates.

Biology Open • Supplementary information

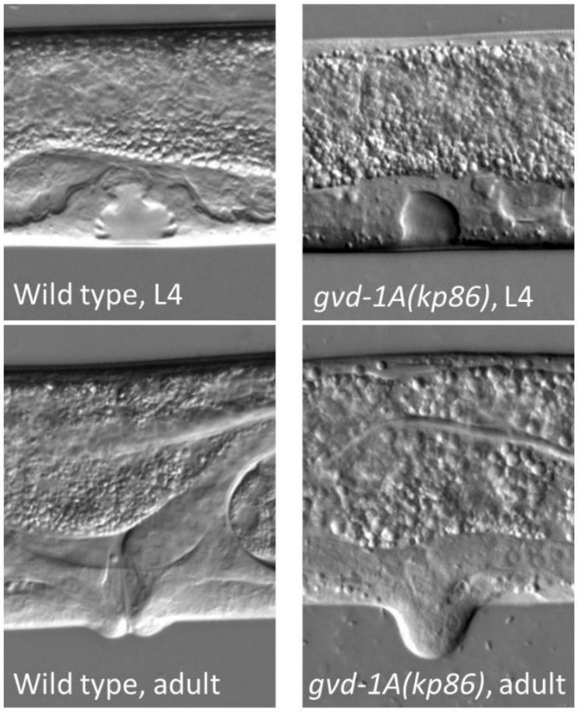

Fig. S18. Differential interference contrast images depicting vulval defects in *gvd- 1A(kp86)* animals. (Top) While the wild-type larva forms characteristic Christmas tree-shaped invagination at the L4 stage, the developing vulva invaginates abnormally in the mutant. (Bottom) *gvd-1A(kp86)* adults form a protruding structure where the wild-type develops a normal vulva.

Biology Open • Supplementary information

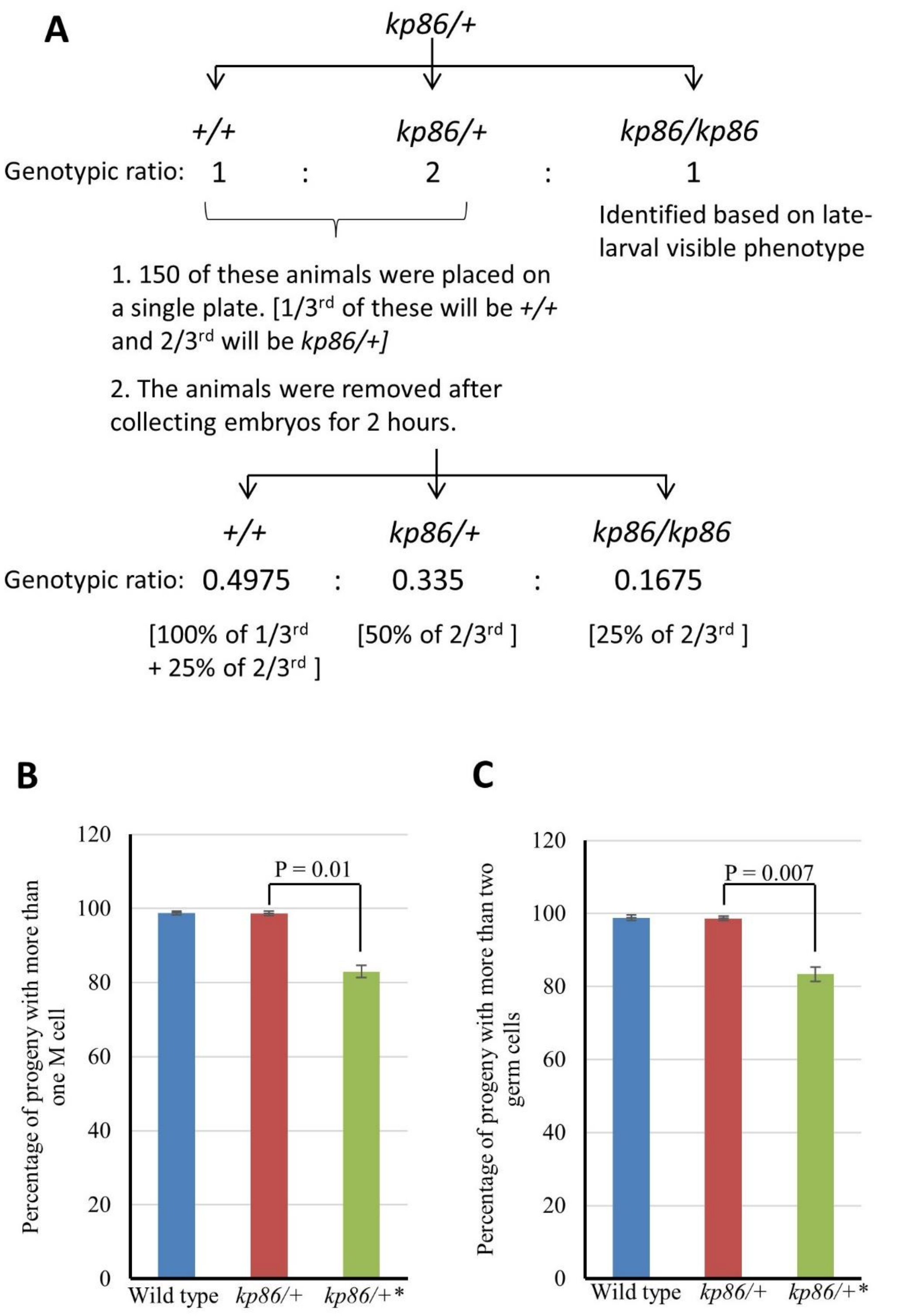

Fig. S19. Cell divisions in the M and germ cell lineages during L1 stage are unaffected in *gvd-1A(kp86)* animals. M cell and PGC descendants were identified using *hlh-8p*::GFP and PGL-1::TagRFP, respectively in the progenies of animals of the indicated genotypes (Harfe et al., 1998; Wan et al., 2019). Since there are no balancers that cover the *gvd-1* region, we

Biology Open • Supplementary information

maintain the *gvd-1A(kp86)* strain as transheterozygote with the mutant allele *tm2403* of the nearby gene *cdc-37* [*gvd-1A(kp86) / cdc-37(tm2403)*]. To avoid the potential influence of *tm2403* [*tm2403* also causes larval defects, although distinct from the *kp86* phenotype], we used *gvd-1A(kp86/+)* animals in this experiment (A). Embryos laid by about 150 wild-type adults or 150 phenotypically wild-type adult progenies of *kp86/+* animals (+/+ and *kp86/+*) during a period of two hours were allowed to hatch and develop for 27.5 hours and 29.5 hours, for counting M descendants and germ cells, respectively. Since *kp86/kp86* homozygous larvae could not be identified at the L1 stage, we allowed some siblings of the counted larvae to reach the L3 stage, at which *kp86/kp86* could be readily identified, and calculated the fraction of the total larvae that were *kp86/kp86*. Because only two-third of the 150 adult progenies of *kp86/+* animals used for embryo collection would be *kp86/+* (see the scheme in A), the expected fraction of *kp86/kp86* among the embryos collected will be 0.17 ($1/4^{th}$ of $2/3^{rd}$). Actual values obtained in the three experiments were 0.15 (n = 1240), 0.15 (n = 1731) and 0.18 (n = 2513) for scoring M cells and 0.18 (n = 1800), 0.15 (n = 2318) and 0.14 (n = 1898) for scoring germ cells. Bars represent the average scores from three experiments. Total number of larvae scored in the three experiments were 814, 845 and 1018 for wild type and 731, 740 and 952 for *kp86/+* (B) and 433, 831 and 1067 for wild type and 482, 989 and 894 for *kp86/+* (C). Error bars represent standard deviations, and the *P* values were calculated by two-tailed Student's *t*-test. *kp86/+** denotes the predicted score if the M cell or PGCs failed to divide in the *kp86/kp86* progenies of *kp86/+* during L1.

Biology Open • Supplementary information

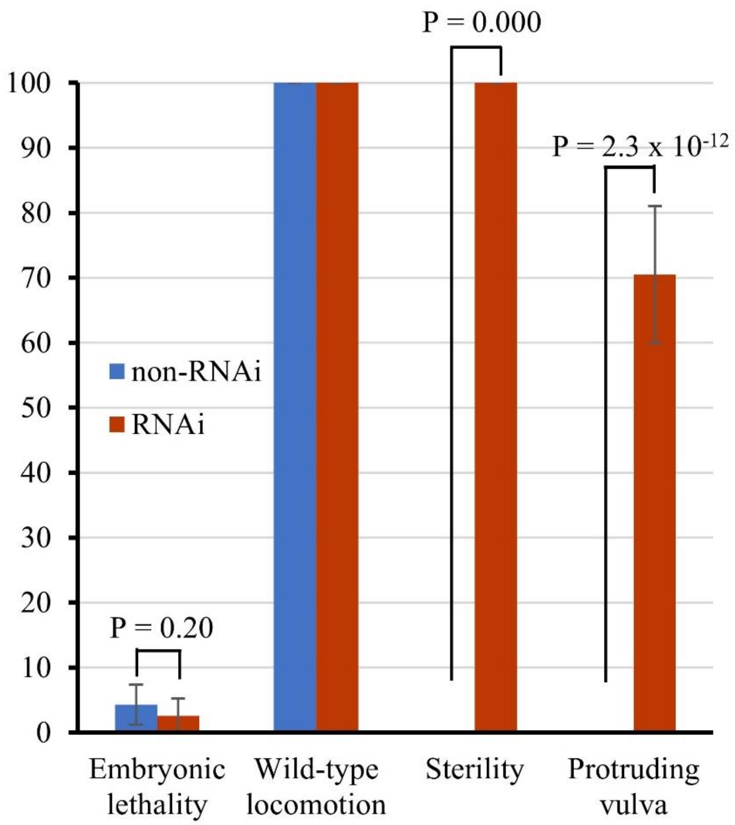

Fig. S20. Depletion of GVD-1 by dsRNA injection does not cause embryonic lethality. Injection of *gvd-1*-specific dsRNA into *gvd-1A(kp86)* animals carrying both *kpIs7* and *kpIs106* transgenes resulted in sterility and protruding vulva, but not embryonic lethality or uncoordinated locomotion, in the progeny. The number of embryos and larvae examined were 430 and 418, respectively. Error bars represent standard deviations, and the *P* values were calculated by two-tailed Student's *t*-test.

Biology Open • Supplementary information

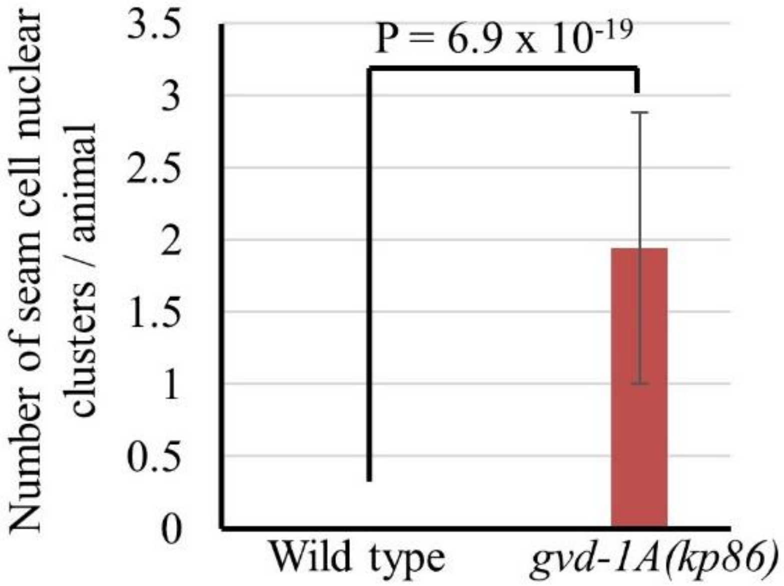

Fig. S21. Nuclei in the seam form clusters in *gvd-1A(kp86)* adults. Nuclei in the syncytial seam were visualized using the SCMp:GFP and the number of nuclear clusters (see Fig. 7A) per animal was counted (n = 35 animals for each genotype). Error bars represent standard deviations, and the *P* values were calculated by two-tailed Student's *t*-test.

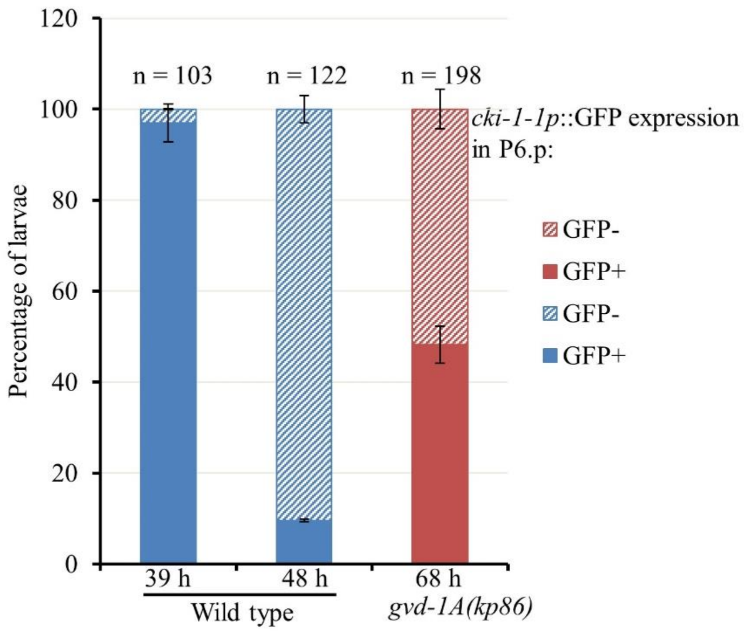

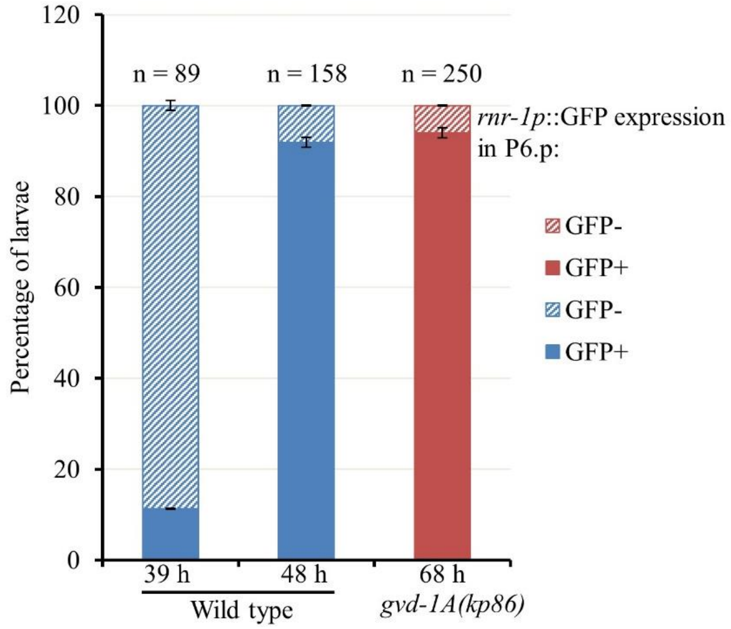

Fig. S22. Expression patterns of *cki-1p*::GFP and *rnr-1p*::GFP P6.p of wild-type and *gvd-1A(kp86)* larvae. The number of larvae that were either positive (GFP+) or negative (GFP-) for the expression of the indicated reporter were counted at the indicated time points post-egg laying and plotted as percentage of the total. The number of larvae examined for *cki-1p:*:GFP expression were 103 and 122 for wild type at 39 hours and 48 hours, respectively, and 198 for *gvd-1A(kp86)* at 68 hours. The number of larvae examined for *rnr-1p:*:GFP expression were 89 and 158 for wild type at 39 hours and 48 hours, respectively, and 250 for *gvd-1A(kp86)* at 68 hours. Error bars represent standard deviations for two replicates for wild type and three replicates for *gvd-1A(kp86)*.

Biology Open • Supplementary information

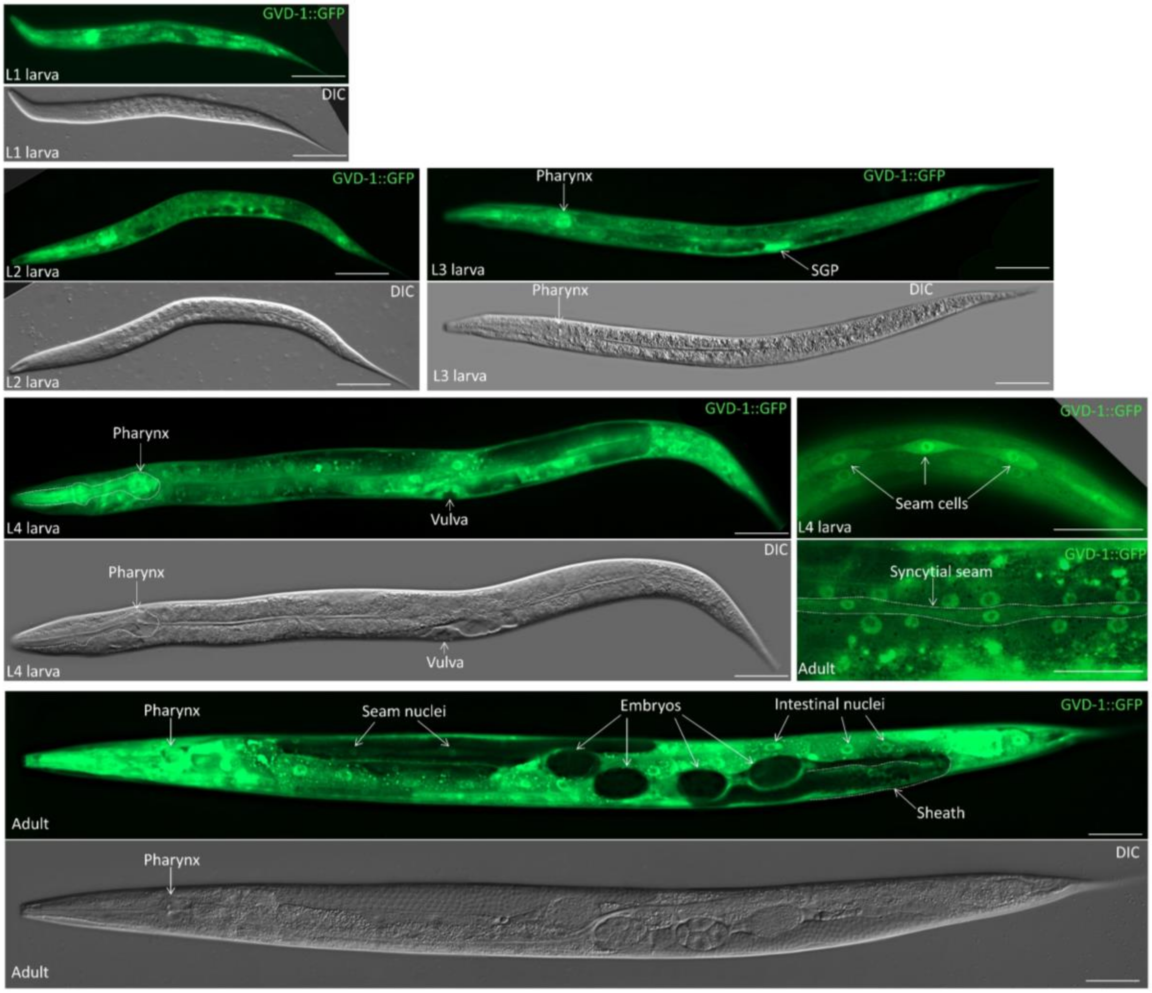

Fig. S23. The *kpIs106* transgene expresses GVD-1::GFP in multiple somatic cell types. Fluorescence (GVD-1::GFP) and the corresponding DIC images of the indicated developmental stages are shown. Certain identifiable structures are labelled. (Third panel, right) Sections of the animal body in which GVD-1::GFP expression in some of the seam cells (top) or part of the syncytial seam (bottom) is visible. Scale bar: 50 μm.

Biology Open • Supplementary information

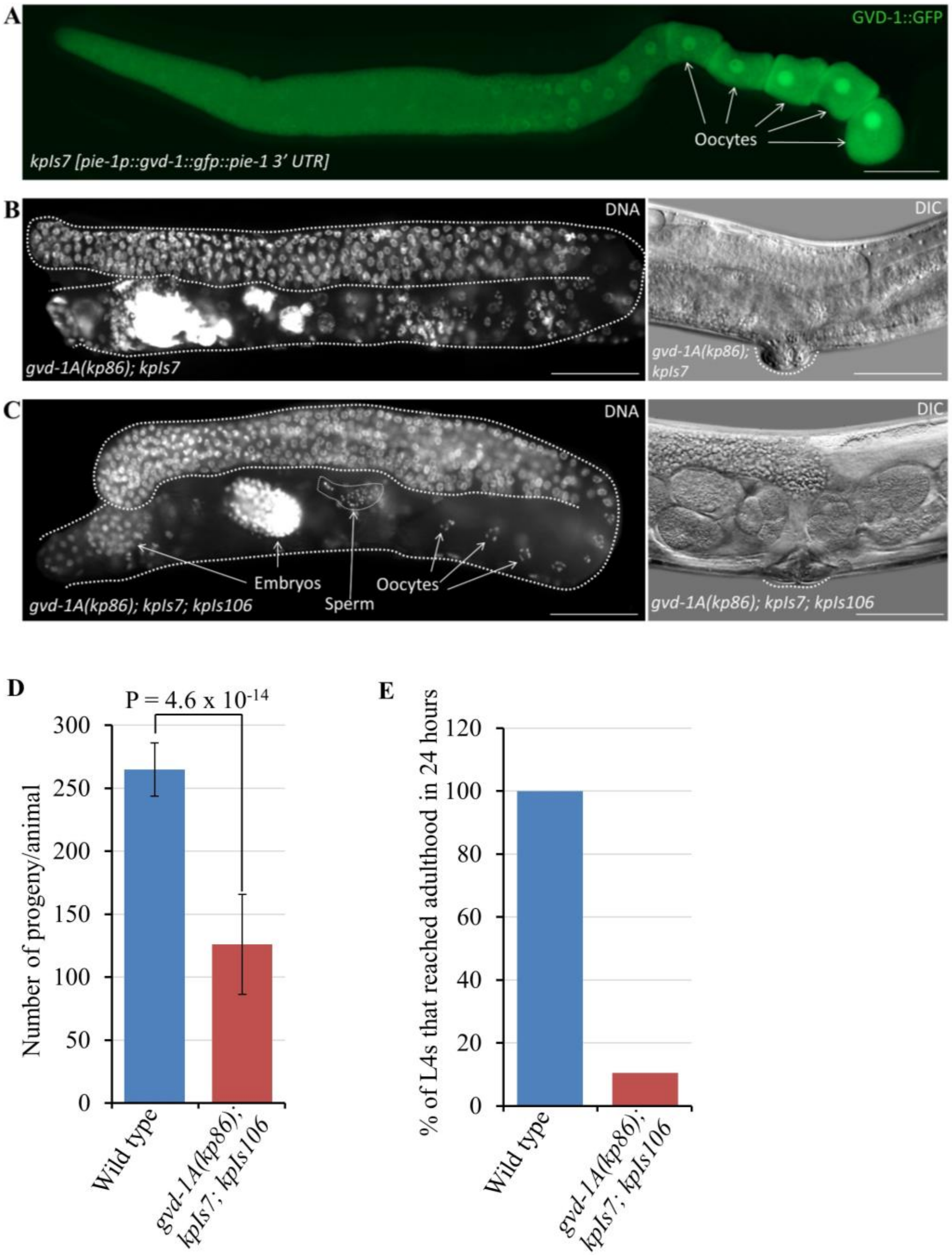

Fig. S24. Expression of GVD-1 in both germ line and soma is essential for fertility. (A) *pie-1* promoter-driven expression of GVD-1::GFP (*kpIs7*) in germ line. GVD-1::GFP is seen throughout the germ line with its nuclear localization more prominent in oocytes. (B) The above transgene partially rescues the germ cell proliferation defect (left; compare with images in Fig. 2) but fails to restore the formation of embryos and normal vulva (right; dashed line) in *gvd-1A(kp86)* animals. (C) Animals expressing GVD-1::GFP in both soma (*kpIs106*; driven by the *gvd-1* promoter) and germ line (driven by the *pie-1* promoter) produced embryos (left) and

Biology Open • Supplementary information

formed normal vulva (right: dashed line). However, the rescue by the transgenes is partial as judged by the brood size (D; n = 17 animals/genotype) and developmental delay (E; n = 19 animals/genotype). Error bars represent standard deviations for two replicates for each genotype, and the *P* value was calculated by two-tailed Student's *t*-test. Scale bar: 50 µm.

Biology Open • Supplementary information

Table S1. Results of the two-factor mapping crosses

Recombination frequency between *kp20* and the marker mutations are listed.

Chromosome	Marker mutation	Recombination frequency	Total number of plates examined
**I**	***dpy-5***	**54 %**	**13**
**II**	***rol-6***	**100 %**	**13**
**III**	***lon-1***	**46 %**	**13**
**IV**	***bli-6***	**73 %**	**26**
**V**	***dpy-11***	**73 %**	**26**
**X**	***lon-2***	**69 %**	**26**

Table S2. Results of the segregation analysis

Genotype	Total number of worms screened	Percentage of sterile worms
puf-8(ok302) unc-4(e120) (uncoordinated locomotion)	515	53.6
puf-8(zh17) rol-6(e187) (“roller” worms)	588	8.8

Table S3. List of genes carrying mutations in the region between -18 and -6 on chromosome II and the effect of RNAi on them

**Serial no.**	**Gene name**	**Genetic position**	**Nature of mutation**	**RNAi phenotype on**	
				puf-8 (ok302)/+ (Number of animals scored)	***puf-8(ok302)*** (Number of animals scored)
1	*gfp* (control RNAi)			100% fertile (200)	100% fertile (200)
2	C50D2.7	-16.19	Missense	100% fertile (200)	100% fertile (200)
**3**	**W10D9.6**	**-15.6**	Missense	**100% fertile (500)**	**99% sterile (718)**
4	K05F6.5	-15.4	Missense	100% fertile (200)	100% fertile (200)
5	T07H3.5	-15.37	Frame-shift	100% fertile (200)	100% fertile (200)
6	F07E5.1	-13.9	Frame-shift	100% fertile (200)	100% fertile (200)
7	F07E5.8	-13.89	Missense	100% fertile (200)	100% fertile (200)
8	ZK1240.8	-13.83	Missense	100% fertile (200)	100% fertile (200)
9	Y110A2AM.1	-9.38	Frame-shift	100% fertile (200)	100% fertile (200)
10	T11F1.8	-9.21	Missense	100% fertile (200)	100% fertile (200)
11	C41H7.1	-9.02	Missense	100% fertile (200)	100% fertile (200)
12	W09G10.1	-6.24	Frame-shift	100% fertile (200)	100% fertile (200)

Table S4. List of *C. elegans* strains used in this study

Strain Name	Genotype	Reference
EG6703	*unc-119(ed3) III; cxTi10816 IV; oxEx1582.*	(Frokjaer-Jensen et al., 2012)
GC678	*tnIs6[lim-7p:GFP; rol-6 (su1006)]; qIs19[lag-2p:GFP; rol-6(su1006)]*	(Killian and Hubbard, 2005)
GC833	*glp-1(ar202) III*	(Pepper et al., 2003)
IT97	*puf-8(zh17) rol-6(e187)/mnC1 II*	This study
IT873	*gvd-1(kp20) puf-8(ok302) unc-4(e120)/mnC1 II*	This study
IT874	*gvd-1(kp20) puf-8(zh17) rol-6(e187) gvd-1(kp20)/mnC1 II*	This study
IT1074	*kpIs7[pie-1p:GVD-1::GFP:pie-1 3'UTR]*	This study
IT1167	*gvd-1A(kp86)/+ II*	This study
IT1179	*gvd-1A(kp86)/+ II; qIs19[lag-2p:GFP; rol-6(su1006)]*	This study
IT1183	*gvd-1A(kp86)/+ II; jcIs1[AJM-1::GFP + unc-29(+) + rol-6(su1006)] IV*	This study
IT1220	*gvd-1A(kp86)/+ II; tnIs6[lim-7p:GFP; rol-6 (su1006)]*	This study
IT1228	*cdc-37(tm2403)/+ II*	Obtained from the Mitani laboratory
IT1231	*gvd-1A(kp86)/cdc-37(tm2403) II*	This study
IT1235	*gvd-1A(kp86)/cdc-37(tm2403) II; syIs59[egl-17p:CFP + unc-119(+)] X*	This study
IT1244	*kpIs106 [gvd-1p:GVD-1::GFP:gvd-1UTR + unc-119(+)] IV*	This study
IT1247	*gvd-1A(kp86)/cdc-37(tm2403) II; kpIs106 IV*	This study
IT1263	*gvd-1A(kp86)/+ II; qyls7(LAM-1::GFP); qyls17(zmp-1p:mCherry)*	This study
IT1264	*gvd-1A(kp86)/cdc-37(tm2403) II; kpIs106 IV; kpIs7*	This study
IT1266	*gvd-1A(kp86)/cdc-37(tm2403) II; kpIs7*	This study
IT1336	*gvd-1A(kp86)/cdc-37(tm2403) II; glp-1(ar202) III; kpIs106 IV*	This study
IT1361	*gvd-1A(kp86)/cdc-37(tm2403) II; wIs51 V*	This study
IT1409	*gvd-1A(kp86)/cdc-37(tm2403) II; maIs103*	This study
IT1424	*jcIs1 [ajm-1::GFP + rol-6(su1006)] IV; wIs51 V [SCMp:GFP + unc-119(+)] V.*	This study

Biology Open • Supplementary information

IT1425	*gvd-1A(kp86)/cdc-37(tm2403) II; jcIs1 [AJM-1::GFP + rol-6(su1006)] IV; wIs51 V [SCMp:GFP + unc-119(+)] V.*	This study
IT1434	*gvd-1A(kp86)/gvd-1A(kp86) II; kpIs106 IV; kpIs7*	This study
IT1450	*gvd-1A(kp86)/cdc-37(tm2403) II; maIs113*	This study
IT1488	*gvd-1A(kp86)/+ II; gg547*	This study
IT1489	*gvd-1A(kp86)/+ II; ayIs6 X*	This study
JH1500	*puf-8(ok302) unc-4(e120)/mnC1 II*	(Subramaniam and Seydoux, 2003)
JR667	*unc-119(e2498::Tc1) III; wIs51 V [SCMp:GFP + unc-119(+)] V*	(Terns et al., 1997)
NK272	*qyls7(LAM-1::GFP); qyls17(zmp-1p:mCherry)*	(Matus et al., 2015; Medwig-Kinney et al., 2020).
PD4666	*ayIs6 [hlh-8p::GFP + dpy-20(+)] X*	(Harfe et al., 1998)
PS3525	*syIs59[egl-17p:CFP + unc-119(+)] X*	(Inoue et al., 2002)
SU93	*jcIs1[AJM-1::GFP + unc-29(+) + rol-6(su1006)] IV*	(Koppen et al., 2001)
VT765	*unc-36(251); maIs103[rnr-1p:GFP + unc-36(+)]*	(Hong et al., 1998)
VT825	*dpy-20(e1282) IV; maIs113[cki-1p:GFP]*	(Hong et al., 1998)
YY967	*pgl-1(gg547[pgl-1::3xflag::tagrfp])*	(Wan et al., 2019)

Biology Open • Supplementary information

Table S5 List of oligonucleotides used in this study

Name	Sequence	Description
KS4769	TTATGGCGGATTTCTTGGAG	PCR primers for amplification of *gvd-1* from cDNA
KS4770	CGAAATTCCCAAAAGCAG	
KS6417	TCTGCATAATACGACTCACTATAGGGTATAGGGAGACCGGCAGATC	PCR primers for *gvd-1* sense RNA template preparation
KS2484	GGTCGACGGTATCGATAAGC	
KS2483	TATAGGGAGACCGGCAGATC	PCR primers for *gvd-1* antisense RNA template preparation
KS6418	TCTGCATAATACGACTCACTATAGGGTCGACGGTATCGATAAGC	
KS4988	GGAGGACTCTGAGCAAAATGATCGAAATATCTGCCGCTAGCTAAACAGACTTCCTAGCCGATCAATTCGAAAAATTT	Repair template for *kp86* edit
KS4996	TCTTGATCGGCTAGGAAGTCTGTT	sgRNA template for *kp86* edit
KS4997	AAACAACAGACTTCCTAGCCGATC	
KS6468	TCTTGCTCATGTGACTGAGCCAGT	sgRNA template (pKS232) for *kp91* edit
KS6469	AAACACTGGCTCAGTCACATGAGC	
KS4670	TCTTGCCTCATGTGACTGAGCCAG	sgRNA template (pKS233) for *kp91* edit
KS4671	AAACCTGGCTCAGTCACATGAGGC	
KS6473	GATGGTTTTATCACAATTCCACACGCTAAATCCTCATGTGACTGAGCCACATGGGAGCCTTGAAATCGTCCATTTTTCCTGAAAAAACCCAAAAATTTCTATG	Repair template for *kp91* edit
KS5071	CGAATTCAGCACGTCATTTTACCTCGTG	Single-worm PCR primers for detecting the *kp86* edit
KS5073	GATCGAAATATCTGCCGCTAGCT	
KS6474	TTCAAGGCTCCCATGTGGCT	Single-worm PCR primers for detecting the *kp91* edit
KS6475	GTACTCTTGGTGCTACAGTA	
KS4780	TCTGCAGGATCCATGGCGGATTTCTTGGAGGACTCT	Forward primer for PCR-amplification of *gvd-1* coding sequences
KS4781	TCTGCAGGATCCAATTTTCAGTCCATTTTTCATGTGATCG	Reverse primer for PCR-amplification of *gvd-1* coding sequences
KS4921	TCTGCACCGCGGGGATCCTCCCATGCCCACTGGCTCAGTCACATGAG	Forward primer for PCR-amplification of *gvd-1* upstream sequences
KS1468	TCTACTAGTATGAGTAAAGGAGAAGAACTTTTCAC	PCR primers for amplification of GFP coding sequences from pKS114
KS3324	TCTACTAGTATGAGTAAAGGAGAAGAACTTTTCAC	
KS4924	TCTGCAGGGCCCATAAATTCCTTTTTTTTTTGTTGATCTCTGAGTATTTAG	PCR primers for amplification of *gvd-1* downstream sequences
KS4940	TCTGCAGGGCCCCAAGCATTATTTCAATTCTCGGAGAATGAAT	

Biology Open • Supplementary information
